# Renal Association Clinical Practice Guideline on Haemodialysis

**DOI:** 10.1186/s12882-019-1527-3

**Published:** 2019-10-17

**Authors:** Damien Ashby, Natalie Borman, James Burton, Richard Corbett, Andrew Davenport, Ken Farrington, Katey Flowers, James Fotheringham, R. N. Andrea Fox, Gail Franklin, Claire Gardiner, R. N. Martin Gerrish, Sharlene Greenwood, Daljit Hothi, Abdul Khares, Pelagia Koufaki, Jeremy Levy, Elizabeth Lindley, Jamie Macdonald, Bruno Mafrici, Andrew Mooney, James Tattersall, Kay Tyerman, Enric Villar, Martin Wilkie

**Affiliations:** 1 Hammersmith Hospital, Imperial College Healthcare NHS Trust, London, England; 2 Wessex Kidney Centre, Portsmouth NHS Trust, Portsmouth, England; 3 University Hospitals of Leicester NHS Trust, Leicester, England; 4 Royal Free London NHS Foundation Trust, London, UK; 5 Lister Hospital, East & North Hertfordshire NHS Trust, Stevenage, England; 6 Sheffield Teaching Hospitals NHS Foundation Trust, Sheffield, England; 7 School of Nursing and Midwifery, University of Sheffield, Sheffield, England; 8 East & North Hertfordshire NHS Trust, Stevenage, England; 9 Leeds Teaching Hospitals NHS Trust, Leeds, UK; 10 United Lincolnshire Hospitals NHS Trust, Lincoln, UK; 11 Renal and Exercise Rehabilitation, King’s College Hospital, London, England; 12 Great Ormond Street Hospital, London, England; 13 Haemodialysis Patient, c/o The Renal Association, Bristol, UK; 14 School of Health Sciences, Queen Margaret University, Edinburgh, Scotland; 15 Department of Renal Medicine, Leeds Teaching Hospitals NHS Trust, Leeds, England; 16 School of Sport, Health and Exercise Sciences, Bangor University, Bangor, UK; 17 Nottingham University Hospitals NHS Trust, Nottingham, UK

## Abstract

This guideline is written primarily for doctors and nurses working in dialysis units and related areas of medicine in the UK, and is an update of a previous version written in 2009. It aims to provide guidance on how to look after patients and how to run dialysis units, and provides standards which units should in general aim to achieve. We would not advise patients to interpret the guideline as a rulebook, but perhaps to answer the question: “what does good quality haemodialysis look like?”

The guideline is split into sections: each begins with a few statements which are graded by strength (1 is a firm recommendation, 2 is more like a sensible suggestion), and the type of research available to back up the statement, ranging from A (good quality trials so we are pretty sure this is right) to D (more like the opinion of experts than known for sure). After the statements there is a short summary explaining why we think this, often including a discussion of some of the most helpful research. There is then a list of the most important medical articles so that you can read further if you want to – most of this is freely available online, at least in summary form.

A few notes on the individual sections:

1. This section is about how much dialysis a patient should have. The effectiveness of dialysis varies between patients because of differences in body size and age etc., so different people need different amounts, and this section gives guidance on what defines “enough” dialysis and how to make sure each person is getting that. Quite a bit of this section is very technical, for example, the term “eKt/V” is often used: this is a calculation based on blood tests before and after dialysis, which measures the effectiveness of a single dialysis session in a particular patient.
2. This section deals with “non-standard” dialysis, which basically means anything other than 3 times per week. For example, a few people need 4 or more sessions per week to keep healthy, and some people are fine with only 2 sessions per week – this is usually people who are older, or those who have only just started dialysis. Special considerations for children and pregnant patients are also covered here.
3. This section deals with membranes (the type of “filter” used in the dialysis machine) and “HDF” (haemodiafiltration) which is a more complex kind of dialysis which some doctors think is better. Studies are still being done, but at the moment we think it’s as good as but not better than regular dialysis.
4. This section deals with fluid removal during dialysis sessions: how to remove enough fluid without causing cramps and low blood pressure. Amongst other recommendations we advise close collaboration with patients over this.
5. This section deals with dialysate, which is the fluid used to “pull” toxins out of the blood (it is sometimes called the “bath”). The level of things like potassium in the dialysate is important, otherwise too much or too little may be removed. There is a section on dialysate buffer (bicarbonate) and also a section on phosphate, which occasionally needs to be added into the dialysate.
6. This section is about anticoagulation (blood thinning) which is needed to stop the circuit from clotting, but sometimes causes side effects.
7. This section is about certain safety aspects of dialysis, not seeking to replace well-established local protocols, but focussing on just a few where we thought some national-level guidance would be useful.
8. This section draws together a few aspects of dialysis which don’t easily fit elsewhere, and which impact on how dialysis feels to patients, rather than the medical outcome, though of course these are linked. This is where home haemodialysis and exercise are covered.

There is an appendix at the end which covers a few aspects in more detail, especially the mathematical ideas. Several aspects of dialysis are not included in this guideline since they are covered elsewhere, often because they are aspects which affect non-dialysis patients too. This includes: anaemia, calcium and bone health, high blood pressure, nutrition, infection control, vascular access, transplant planning, and when dialysis should be started.

## Introduction

Haemodialysis continues to expand in the UK with over 25 000 patients now being treated, representing a 10% increase since publication of the previous Renal Association guideline for haemodialysis. In addition the patient group continues to develop: the typical patient is now 67 years old with a median history of 3.2 years on renal replacement therapy. The authors of this guideline aimed principally to update the previous guideline according to the latest research and experience, but also to expand the scope into areas not previously covered but relevant to haemodialysis practice.

The guideline was written collaboratively: lead and co-authors for each section conducted literature reviews and wrote first drafts of the statements and rationale. Feedback and discussion were provided by all authors via email exchanges and meetings, revised versions were produced with editorial input from the chair, and these were subsequently agreed by all authors. Two current haemodialysis patients gave advice on tone and readability.

Systematic literature searches were undertaken by lead authors to identify all relevant evidence published up until the end of June 2018. Compound search terms were used which included a dialysis identifier (hemodialysis[tiab] OR haemodialysis[tiab] OR dialysis[tiab]) followed by title/abstract-filtered topic terms (“dialysis dose”, Kt/V, augmented, intensive, conservative, incremental, pregnancy, membrane, hydration, “dry weight”, “fluid overload”, dialysate, potassium, bicarbonate, buffer, phosphate, “dialyser reaction”, hypersensitivity, “blood loss”, “needle dislodgment”, exsanguination, “home haemodialysis”, “nocturnal haemodialysis”, exercise, “physical training”) followed by negative terms (e.g. to exclude animal studies and acute kidney injury) finally with date and language restrictions (“1990/01/01”[dp]: “3000”[dp] AND english[lang]). Searches were conducted in MEDLINE, PUBMED, Embase, and The Cochrane Library, and supplemented with papers handpicked from the reference lists of review papers.

The strengths of the recommendations and the level of supporting evidence are coded as previously using the Modified GRADE system.

There are a few changes in scope, for example dialysis water treatment is now covered in another guideline, as are many aspects of dialysis, including:

- Planning, initiation & withdrawal of Renal Replacement Therapy
- Vascular Access for Haemodialysis
- Cardiovascular Disease
- Blood Borne Viruses
- Assessment of the Potential Kidney Transplant Recipient
- Nutrition
- Anaemia
- CKD-Mineral and Bone Disorder
- Water Treatment Facilities, Dialysis Water and Dialysis Fluid Quality

We have removed the section on targets for blood testing since these are better covered in other guidelines, and have not covered infrastructure or workforce since these will be addressed separately by the Renal Association in a different format.

However, in most ways the update is broader than previous versions. For example, new sections have been written covering fluid management (surely an essential topic but not really covered previously or elsewhere) and dialysate (often underestimated in importance). In other areas this update seems to make no substantial change to previous guidance (as with dialysis dose, for example, where the literature remains dominated by previous large trials), however whilst key concepts remain valid, their understanding has developed, and the guideline aims to provide greater context, encouraging a more holistic interpretation.

Discussions about dialysis often become overly technical – these concepts are important but hard to fit into a narrative so we have moved a few aspects into the appendix, where we aim to provide simplified summaries. We have tried to maintain a high standard of readability since conceptual understanding is the key goal, and as the guideline is not intended to replace review articles or original papers, it seems correct to favour readability over detail.

## Summary of clinical practice guidelines

### Dialysis dose in thrice weekly dialysis schedules

We recommend eKt/V as the most clinically valid small-solute measure of dialysis dose, and recommend monitoring of dialysis dose on a monthly basis for the majority of centre-based dialysis patients. [1B]

We recommend targeting dialysis dose to achieve consistently a minimum eKt/V of 1.2 for thrice weekly patients, in the absence of a measured contribution from residual function. [1B]

We recommend a minimum of 12 hours per week for the majority of thrice weekly patients with minimal residual function. [1B]

### Non-standard schedules (Guidelines 2.1 – 2.4)

#### Guideline 2.1 - Augmented schedules

We suggest offering an augmented schedule to patients who are unable to achieve adequacy targets or fluid control on a standard thrice weekly schedule. [2B]

We suggest that relative contraindications to augmented schedules should be considered, such as significant residual function or problematic fistula access. [2C]

#### Guideline 2.2 - Incremental schedules

We suggest that lower haemodialysis dose targets may be optimal in patients with significant residual renal function. [2D]

We recommend that residual renal function should be quantified intermittently in patients on incremental dialysis schedules. [1D]

#### Guideline 2.3 - Conservative schedules

We suggest that lower haemodialysis dose targets may be optimal when quality of life is the primary goal of treatment, rather than longevity. [2D]

#### Guideline 2.4 - Paediatric schedules

In children and adolescents we recommend an approach to the assessment of dialysis adequacy which goes beyond biochemical targets, incorporating clinical goals such as growth, bone health, cardiac function and quality of life. [1C]

We recommend targeting dialysis dose to achieve a minimum eKt/V of 1.2 for thrice weekly patients, or a standardized Kt/V of 2.2 for those on augmented schedules. [1C]

We suggest an augmented schedule for children on predominantly liquid nutrition, and those with ventricular systolic dysfunction. [2D]

We recommend a blood flow rate of 5-7ml/kg/min for the majority of patients, using consumables appropriate to body size, with extracorporeal volume less than 10% of the patient’s blood volume. [1C]

#### Guideline 2.5 -Schedules during pregnancy

We recommend counselling women of reproductive age who are receiving or anticipating dialysis, so that they are aware of the interactions between renal replacement therapies and pregnancy which may impact on family planning and modality decisions. [1D]

For dialysis patients wishing to continue their pregnancy, we recommend changing as early as possible to an individualised, augmented haemodialysis schedule. For those with minimal residual function this should be at least 20 hours per week, delivered over at least 6 sessions. [1B]

We recommend an individualised dialysate prescription appropriate to the dialysis schedule and biochemistry results, anticipating the frequent need for a high potassium / low bicarbonate dialysate, supplemented with phosphate. [1C]

We suggest an individualised fluid management protocol, with low ultrafiltration rates and regular clinical assessment, anticipating the typical change in weight during pregnancy. [2C]

### Membrane flux and haemodiafiltration

We recommend that patients with minimal residual function should be treated with high-flux dialysers. [1B]

We suggest that haemodiafiltration may be considered as a treatment for intra-dialytic hypotension refractory to other measures, and for dialysis patients with favourable prognosis who are unable or unlikely to be transplanted. [2B]

### Fluid in haemodialysis (Guidelines 4.1 – 4.2)

#### Guideline 4.1 - Fluid assessment and management in adults

We recommend assessment of fluid status when prompted by clinical circumstances, and on a quarterly basis for stable patients. [1C]

We suggest a multidisciplinary approach to fluid assessment, with patient involvement and the adoption of patient-friendly terminology such as “target weight”, “fluid gain” and “over-hydration”. [2D]

We recommend clinical assessment of fluid status on a monthly basis for the majority of patients. [1C]

We suggest supplementing clinical assessment of fluid status with a validated objective measurement, such as bioimpedance, at regular intervals, when clinical assessment is unclear, and following an intercurrent illness. [2C]

We recommend a dialysate temperature not greater than 36'C if standardised. [1C]

We recommend avoiding excessive ultrafiltration rates by addressing fluid gains, accepting staged achievement of target weight, or using an augmented schedule, as necessary. [1B]

We recommend prompt nursing intervention to restore haemodynamic stability in symptomatic / severe intradialytic hypotension, with such interventions leading to clinical review. [1C]

#### Guideline 4.2 - Paediatric fluid considerations

In growing children we recommend clinical assessment of fluid status and target weight, and dietetic assessment, at least monthly. [1C]

We suggest supplementing clinical assessment with a validated objective measure of fluid status such as bioimpedance, on a monthly basis or more frequently during periods of rapid growth or illness. [2C]

We recommend regular assessment of ultrafiltration tolerance, using extended times to avoid excessive ultrafiltration rates. [1D]

### Dialysate (Guidelines 5.1 – 5.4)

#### Guideline 5.1 -Selection of dialysate potassium

We recommend an optimal pre-dialysis serum potassium in the range 4.0–6.0mmol/L, remembering to consider measurement errors (e.g. due to haemolysis) when interpreting levels. [1B]

We suggest choosing dialysate potassium between 1.0 and 3.0mmol/L for the majority of patients, using an individualised approach, in general using the highest dialysate potassium that is sufficient to control pre-dialysis hyperkalaemia. [2C]

We suggest a combined approach to managing hyperkalaemia, which may include decreasing dialysate potassium and/or other measures, including dietary advice, medication review and increased dialysis frequency. [2D]

#### Guideline 5.2 - Selection of dialysate buffer

We recommend an optimal pre-dialysis serum bicarbonate in the range 18.0-26.0mmo/L, remembering to consider measurement errors (e.g. due to exposure to air) when interpreting levels. [1C]

We suggest the term ‘dialysate buffer’ rather than ‘dialysate bicarbonate’ to avoid confusion arising from differences in manufacturers’ terminology. [2C]

We suggest choosing dialysate buffer below or equal to 37.0mEq/L for the majority of patients, using a standardised or individualised approach. [2C]

We suggest a combined approach to abnormal pre-dialysis serum bicarbonate, which may include increasing dialysis dose, oral bicarbonate, nutritional support, or individualising dialysate buffer. [2D]

#### Guideline 5.3 - Supplementation of dialysate with phosphate

We suggest considering supplementation of the dialysate with phosphate in patients on augmented dialysis schedules. [2D]

#### Guideline 5.4 - Paediatric dialysate considerations

We recommend individualisation of dialysate electrolyte concentrations, including potassium, buffer and calcium. [1C]

We suggest an individualised dialysate temperature, between core temperature and 0.5°C below, with monitoring of intradialytic core temperature for neonates and smaller children. [2D]

### Anticoagulation

We recommend that patients without increased bleeding risk should be given unfractionated or low-molecular-weight heparin during dialysis to reduce clotting of the extracorporeal system. [1A]

We recommend that systemic anticoagulation should be omitted or minimised in patients with increased bleeding risk. [1C]

We recommend that patients with heparin allergies should be prescribed a non-heparin form of anticoagulation. [1A]

### Adverse events during dialysis (Guidelines 7.1 – 7.3)

#### Guideline 7.1 - Routine blood loss

We suggest that during washback, dialysis lines and dialyser are observed to ensure residual blood loss is kept to a minimum. [2C]

#### Guideline 7.2 - Disconnection haemorrhage

We recommend maintaining awareness of the risk of disconnection, the limitations of pressure alarms, and importance of direct observation, through a program of education, including patients and carers. [1D]

We suggest regular assessment of individual risk, so that high risk patients can have enhanced monitoring, which could include specific devices. [2B]

#### Guideline 7.3 - Immune reactions during dialysis

We recommend that dialysis staff should be aware of the features and management of dialysis reactions, and should have access to a range of dialyser types. [1C]

### Patient experience of dialysis (Guidelines 8.1 – 8.4)

#### Guideline 8.1 - Home haemodialysis

We recommend that home haemodialysis should be available in all units as part of a comprehensive renal replacement therapy programme. [1A]

We suggest training patients and/or care partners to achieve a defined set of competencies, using an individualised approach to training method and speed. [2D]

We suggest units form a contract with patients outlining responsibilities, including an agreement to dialyse as per prescription and trained technique, and including a policy for re-imbursement of directly arising patient costs. [2D]

We suggest supporting patients with a specific team including nephrologists, technicians, and nurses, with rapid access to dialysis in-centre when required. [2C]

We suggest an agreed individualised prescription for home haemodialysis, taking into account lifestyle goals, with the same dose and time target considerations as centre-based patients. [2C]

We recommend enhanced safety measures for patients who dialyse alone or overnight, and an enhanced risk assessment for patients with blood-borne viruses. [1C]

#### Guideline 8.2 - Shared haemodialysis care

We suggest that all centre-based haemodialysis patients should have opportunity and encouragement to learn aspects of their dialysis treatment, and take an active role in their care. [2D]

#### Guideline 8.3 - Intradialytic exercise

We recommend that intradialytic exercise should be available in all units, as a treatment for enhancing physical functioning, in patients without contraindications. [1B]

We suggest that intradialytic exercise be considered as a method of enhancing quality of life. [2C]

We suggest that exercise regimes be devised by appropriately trained staff. [2C]

#### Guideline 8.4 - Dialysis experience for children and adolescents

We recommend that haemodialysis for children and adolescents should be delivered in a dedicated paediatric dialysis centre or at home, with the involvement of a paediatric multidisciplinary team. [1C]

We recommend that adolescents should commence an active transition programme by 14 years, or at the time of presentation in those already over 14. [1D]

## Summary of audit measures

- **Audit Measure 1:** Amongst thrice-weekly patients on dialysis for more than a year, the median eKt/V, and proportion achieving eKt/V at least 1.2.
- **Audit Measure 2:** Amongst thrice-weekly patients on dialysis for more than a year, the median dialysis time per week, and proportion receiving at least 12 hours.
- **Audit Measure 3:** The proportion of patients dialysing 4 or more times per week (either in-centre or at home).
- **Audit Measure 4:** The proportion of patients dialysing less than 3 times per week, separated into: (a) patients in their first year of dialysis, and (b) patients on dialysis for more than a year.
- **Audit Measure 5:** The median ultrafiltration rate, and proportion of patients with residual kidney function (Kru > 2ml/min, or urine volume > 500ml/d), separated into: (a) patients in their first year of dialysis, and (b) patients on dialysis for more than a year.
- **Audit Measure 6:** The proportion of patients receiving haemodiafiltration, and the median convection volume in this group.
- **Audit Measure 7:** The most commonly used dialysate sodium level, and proportion of patients using this dialysate sodium level.
- **Audit Measure 8:** The availability of an objective tool for fluid state assessment, the type of tool used most commonly, and the proportion of patients assessed with an objective tool during the last year.
- **Audit Measure 9:** The median pre-dialysis serum potassium, and proportion of patients arriving with average potassium over 6.0mmol/l, and proportion with average under 4.0mmol/l.
- **Audit Measure 10:** The proportion of patients using a dialysate potassium level in the following categories: less than 2.0, 2.0, and more than 2.0mmol/l.
- **Audit Measure 11:** The number of disconnection haemorrhage events each year.
- **Audit Measure 12:** The proportion of haemodialysis patients having all or most of their dialysis at home.
- **Audit Measure 13:** The proportion of in-centre patients recognised as engaging in “Shared Care”.
- **Audit Measure 14:** The availability of a program for intra-dialytic exercise, the resource available (equipment, physiotherapist time), and the proportion of in-centre patients engaging with regular intra-dialytic exercise.

## Rationale for Clinical Practice Guidelines

### Dialysis dose in thrice weekly dialysis schedules

We recommend eKt/V as the most clinically valid small-solute measure of dialysis dose, and recommend monitoring of dialysis dose on a monthly basis for the majority of centre-based dialysis patients. [1B]

We recommend targeting dialysis dose to achieve consistently a minimum eKt/V over 1.2 for thrice weekly patients, in the absence of a measured contribution from residual function. [1B]

We recommend a minimum of 12 hours per week for the majority of thrice weekly patients with minimal residual function. This may lead to higher than minimum eKt/V in smaller adult patients which is appropriate. [1B]

#### Rationale

Dialysis adequacy encompasses concepts including the clinical assessment of general wellbeing, fluid status, and control of laboratory parameters, along with quantification of the dose of dialysis provided.

The purpose of dialysis is to provide enough removal of uraemic solutes and fluid that accumulate in kidney failure to maintain health and quality of life: more specific goals include control of uraemic symptoms, maintenance of safe electrolyte levels, prevention of nutritional decline, and optimum long term mortality. Whilst the earlier items in this list are readily assessed over a short time scale, concepts of dialysis dose are required to define the amount of dialysis likely to achieve longer term goals of treatment.

Due to the simplicity and low cost of measurement of urea in blood, measurement of dialysis adequacy has historically focused on clearance of small solutes, represented by urea. Concentration of a range of uraemic toxins of larger size (e.g. β-2 microglobulin) is likely to be important, but their measurement is not commonly performed. Use of thrice weekly haemodialysis schedules emerged from the realisation during the early era of haemodialysis treatment that once or twice-weekly haemodialysis schedules in patients with minimal residual function was insufficient to control the symptoms and complications of severe uraemia.

Most research on dialysis dose is therefore based on urea clearance, in patients on a thrice weekly schedule.

Urea clearance may be calculated by three methods in common use: Urea Reduction Ratio, and the ‘single pool’ and ‘equilibrated’ formulas for Kt/V. Kt/V is less commonly calculated by Urea Kinetic Modelling - these methods are summarised mathematically in Appendix 1. The diversity of methods can lead to duplication of effort, confusion over the meaning of targets, and impedes comparison between centres, so a single widely used method would be desirable. As the most adjusted method, and the one which has been most commonly validated in outcome literature, eKt/V appears to be optimum, and we have therefore given dose targets in terms of eKt/V. Equivalent targets using other methods may be derived for individual patients depending on their dialysis duration and fluid removal.

The literature on clinical outcome at different doses of dialysis is dominated by two randomised studies. The National Cooperative Dialysis Study (NCDS) was the landmark study which led to the concept of a threshold dialysis dose above which treatment was adequate, as well as the establishment of Kt/V as the accepted index of dialysis dose. Reporting in 1981, the study randomised 151 patients in a 2x2 design to high vs low time-averaged urea, and short vs long dialysis duration - the key finding was a lower rate of treatment failure (death or hospital admission) in the low urea (high dialysis dose) group [1]. When reanalysing the group with the newly proposed Kt/V measure, a clear threshold effect appeared, with Kt/V defining the watershed between ‘adequate’ and inadequate dialysis (Kt/V over vs under 1.0) [2].

A large number of observational studies subsequently reported an association between higher dialysis doses (beyond merely achieving the NCDS threshold) and improved survival [3–6], and this was tested in the HEMO study. Reporting in 2002 the HEMO study randomised 1846 patients in another 2x2 design to high vs standard dialysis dose (eKt/V 1.45 vs 1.05) and high vs low flux [7]. Over 2.8 years follow-up with groups well separated in terms of achieved eKt/V (1.53 vs 1.16), higher dose provided no benefit in terms of survival or a number of secondary endpoints.

The basic concepts of these studies have not been superseded, hence the recommendation for dialysis dose (eKt/V > 1.2) is based largely on the eKt/V achieved in the standard dose group of the HEMO study. Alternative measures such as URR or spKt/V may be more familiar to some clinicians and equally useful for the majority of patients. Equivalent thresholds using these parameters are approximate since they vary between patients, but the differences are small: Appendix 1 summarises the mathematics behind these concepts.

Whether ‘adequate’ dialysis is the same for all patients or whether dose should be individualised is unclear, but the latter view is supported by several studies suggesting that gender and body size may affect the optimum dialysis dose [8–10]. Observational studies suggest that dialysis dose is more strongly related to survival in women than men, and when the HEMO study analysis is restricted to women, the high dose group show significantly improved survival. The reason for this interaction between gender and optimum eKt/V is unknown, but may be due to the scaling parameter ‘V’, which is lower in women and in less muscular patients, and is an independent predictor of survival. Alternative scaling factors such as body surface area, have been suggested [11–14], but none is in widespread use, and the collinearity between different body size parameters makes analyses difficult to interpret, but it seems likely that the optimum Kt/V may be higher than 1.2 in women and smaller patients, without a clear definition of ‘small’ [15].

#### Dialysis time

The optimum treatment duration for thrice weekly haemodialysis is slightly less clear, since it is difficult to separate the effect of treatment time from dialysis dose [16].

The evolution of dialysis technology has made dialysis dose targets achievable over short dialysis sessions. However, there are uraemic solutes other than urea, such as phosphate and β2-microglobulin, which are also important predictors of outcome, and which are inefficiently removed by dialysis [17, 18]. Extending dialysis duration increases the removal of these highly sequestered and larger molecules, independent of any change in small solute clearance [19, 20]. In the other part of its 2x2 design, the NCDS study also compared session duration (4.5-5.0 vs 2.5-3.0 hours) and although standard significance ‘level’ was not achieved (p=0.06), showed reduced treatment failure in the longer session group [1].

Most observational studies also report improved outcomes with longer treatment times. Low mortality rates were reported from Tassin with 8 hour overnight dialysis, attributed to improved blood pressure control and slower ultrafiltration [21, 22], and lower mortality is associated with longer treatment times in national registry studies (over vs under 3.5 hours in US patients [23], and over vs under 4.5 hours in Australia [24]). The international DOPPS study examined the effect of treatment time whilst controlling for confounders using standard regression and instrumental variable approaches, concluding that patients with the longest treatment time (at least 4 hours) had the lowest risk for all-cause and cardiovascular mortality [25]. Other clinical markers such as blood pressure, anaemia and phosphate control were also improved.

Whilst recognising the limitations of observational studies, a minimum duration for optimum dialysis clearly exists, and is most likely close to 4 hours, at least for patients with minimal residual kidney function. A duration threshold may lead to higher than minimum eKt/V in smaller adult patients, which is appropriate since optimal Kt/V may be higher in this group.

#### Summary

Optimal outcomes in patients on thrice weekly dialysis are achieved with sessions of at least 4 hours, providing eKt/V at least 1.2. Regular monitoring is strongly recommended, and this occurs monthly in the majority of units.

Under achievement may be addressed by attention to vascular access [26], session duration [27], blood or dialysate flow [28–30], dialyser efficiency [31] or anticoagulation [32], and in some patients under achievement may suggest the need for an augmented schedule. Achievement of these targets does not guarantee optimal outcome, with eKt/V being unaffected by missed sessions, for example.

These dose targets apply to thrice weekly patients, with minimal residual function, for whom survival duration is a primary treatment goal. There are specific clinical scenarios and different patient values for which it may be appropriate to adjust or disregard numeric targets for dialysis dose.

### Non-standard schedules (Guidelines 2.1 – 2.4)

#### Guideline 2.1 - Augmented schedules

We suggest offering an augmented schedule to patients who are unable to achieve adequacy targets or fluid control on a standard thrice weekly schedule. [2B]

We suggest that relative contraindications to augmented schedules should be considered, such as significant residual function or problematic fistula access. [2C]

#### Rationale

Dialysis dose on a thrice weekly schedule is limited by patient tolerance and the necessity to utilise ‘slots’ efficiently, so that sessions over 5 hours are very uncommon. ‘Augmented’ in this guideline refers to increased frequency (4-6 sessions per week) or thrice weekly dialysis totalling more than 15 hours per week. The latter is usually delivered nocturnally when in-centre, but both are often delivered in the context of home haemodialysis where much of the evidence regarding augmented dialysis schedules has been obtained.

Augmented schedules have been assessed in four randomised studies [20, 33–35], one interventional study with matched controls [36], and a handful of observational studies. Evidence of clinical benefit limited to interventional studies is summarised below, with studies divided into three groups for ease of discussion, according to the type of augmented schedule [20, 33–37]. A fourth group of augmented schedules which might be termed ‘modestly frequent’ (4 or 5 sessions per week, of up to 4 hours each) is poorly represented in studies.

**Table:** 

Group	Frequent nocturnal	Short daily	Nocturnal
Definition	> 6 x/week > 6 hours	> 6 x/week < 4 hours	3 x/week > 6 hours
Lead author / study type (patient number in intervention group)	Culleton / RCT (26) Rocco / RCT (45)	Chertow / RCT (125)	Ok / NRI (247) Ipema (metanalysis)
Left ventricular mass	Decreased (Culleton) No change (Rocco)	Decreased	
Blood pressure	Improved	Improved	Improved
Hyperphosphatemia	Improved	Improved	Improved
Nutritional status			Improved
Composite health score / quality of life	No change	Improved	

*Abbreviations*: *RCT* randomized controlled trial, *NRI* non-randomised intervention

Where assessed, improvements in depression, cognition or anaemia parameters were generally not seen in these studies, although improvements in these aspects have been reported in a number of observational studies.

Quality of life is an important outcome since the intervention clearly involves increased treatment burden. Observational studies suggest that quality of life of life is improved in daily dialysis by approximately 6%, whereas nocturnal schedules have not been show to improve quality of life [38–40].

The randomised studies were not designed primarily to assess mortality within the study period, but two of these published mortality results with follow-up extended by approximately 2.5 years [41, 42], and mortality effects have also been reported in other types of study. Findings have been surprisingly inconsistent, however, and are summarised in the table below [36, 41–45].

**Table:** 

Group	Frequent nocturnal	Short daily	Nocturnal
Definition	> 6 x/week > 6 hours	> 6 x/week < 4 hours	3 x/week > 6 hours
Lead author / study type (patient number on augmented schedule)	Rocco / RCT (45)	Chertow / RCT (125) Marshall / OS (?) Suri / OS (318)	Ok / NRI (247) Rivara / OS (1206)
Hazard ratio for mortality (less than 1.0 favours augmented schedule)	3.88	0.54 1.00 / 0.41 (unit / home) 1.60	0.28 0.67

Abbreviations: *RCT* randomized controlled trial, *NRI* non-randomised intervention, *OS* observational study

Authors stress that clinical trials of more intensive dialysis were not designed to evaluate mortality, and that observational analyses often employ statistical techniques which do not adequately address the time-varying nature of the risk factors associated with both the initiation of augmented dialysis and mortality.

The larger randomised trials of augmented schedules have also identified potential harms, for example reducing residual function, an important determinant of survival on haemodialysis. In patients who had significant residual function at enrolment, both frequent nocturnal and short daily dialysis led to a more rapid decline in function compared to control groups [46]. Intervention patients had a shorter time to first vascular access intervention, and there were small increases in the burden on carers, as perceived by patients, though the authors highlight that carers themselves were not assessed [47].

Taken together these studies suggest equivalent mortality and modest improvement in some dialysis-related conditions, offset by increased treatment burden and possible harms to vascular access and residual function. Whilst there is no overall advantage for the average patient these studies do suggest specific groups who would be expected to benefit. For example, adequacy targets could certainly be achieved in those still unable to, despite a reasonably long thrice weekly schedule. Similarly, patients failing to achieve fluid control are likely to benefit from an increase in dialysis frequency - this might include those with resistant hypertension, intra-dialytic hypotension, and those with weekend admissions to hospital. The latter group are the obvious contributors to the excess mortality of the two-day dialysis gap, and may have the most to gain from an increase in dialysis frequency. The augmentation of dialysis in these settings should be aimed at achieving a specific purpose, and it is likely that a fourth session per week would be sufficient in many cases.

In conclusion, augmented schedules offer no clear advantage for the majority of patients, but should be considered as a treatment option for those patients whose adequacy or fluid control targets are not met with a standard schedule. A modestly augmented schedule would be sufficient in the majority of these patients.

#### Guideline 2.2 - Incremental schedules

We suggest that lower haemodialysis dose targets may be optimal in patients with significant residual renal function. [2D]

We recommend that residual renal function should be quantified intermittently in patients on incremental dialysis schedules. [1D]

#### Rationale

**Fig. 1:**
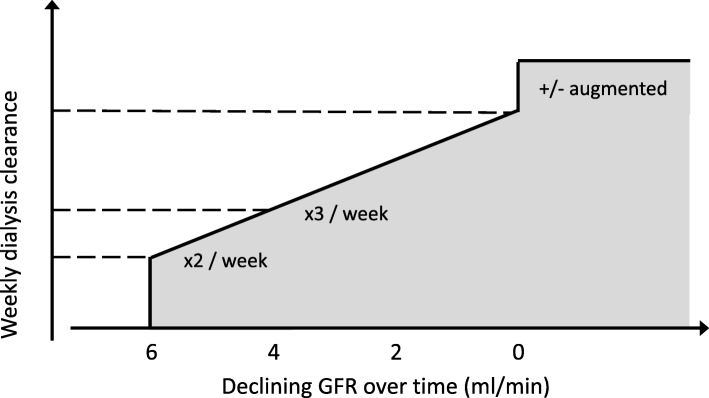
Schematic to illustrate principle of incremental haemodialysis (numbers only as examples)

Incremental haemodialysis is based on the common sense concept that the amount of dialysis required for optimal outcome differs between those with significant residual function and those without. The latter group however is larger, and makes up the majority in studies of dose and outcome, which therefore may not be applicable in the former group. Optimal dialysis dose is therefore not fixed but dependent on the level of residual kidney function, and the prescribed schedule may therefore be reduced in frequency or dose in this setting. The practice of incremental haemodialysis is consistent with a concept of progressively increasing therapy over time, which may include augmented schedules at a later stage (Fig. 1).

Less frequent and reduced dose dialysis practices co-evolved along with standard thrice-weekly schedules: reference is made to twice weekly dialysis in observational studies from the 1990s and in the 1997 KDOQI guidelines [48]. For example, in an observational study of 15 000 American patients published in 1999, Hanson reported twice weekly schedules in 6.1% of patients during their first year, and 2.7% of patients thereafter [49]. Outcomes were at least as good, and in fact a mortality advantage was observed with twice weekly schedules, most likely due to differences in baseline factors: no mortality difference was seen after adjustment for the level of residual function at dialysis initiation.

The non-inferiority of twice weekly schedules in selected patients has been further supported by more recent studies. In a Thai study of 500 twice-weekly patients Panaput reported equivalent mortality and hospitalisation over the next year [50], and in a propensity-matched Korean study of 300 patients followed for one year, Park reported equivalent mortality and improved quality of life with schedules less than thrice-weekly [51]. Non-inferiority of clinical outcome with reduced treatment burden therefore provides a powerful argument in favour of incremental schedules, but additional benefits may exist: incremental haemodialysis schedules have also been associated in some observational studies with reduced decline in residual kidney function [52, 53].

Preservation of residual function is of clinical importance since it provides significant solute and fluid removal, and is associated with improved quality of life and survival [54].

The literature on incremental schedules is limited in particular by its observational nature, with inherent problems of selection and lead-time bias. Variation also exists in the definition of incremental dialysis, which is frequently defined as twice-weekly, without reference to residual function. Clinician bias may also be important: clinicians working in the 1990s will remember twice-weekly schedules principally as a resource-sparing exercise, and even in modern series, financially constraints play a part in their use [55].

Patient selection is therefore crucial: factors currently associated with reduced schedule use in a large Chinese study include early vintage, female sex and minimal comorbidity [56]. And the level of residual function appears perhaps unsurprisingly to be the most important factor: in a large American study in which 350 twice-weekly patients were matched with a thrice-weekly group, twice-weekly schedules yielded equivalent one year outcome in many, but were clearly inferior in those with the poorest residual function (clearance less than 3ml/min/1.73m2) [57]. Those with residual clearance of 3ml/min or less may still be suitable for a thrice-weekly incremental schedule (i.e. with dose target less than Kt/V 1.2 and/or less than 4 hours).

The use of incremental haemodialysis therefore requires regular monitoring of residual function, with function reassessed after major intercurrent illness [58]. Suitable patients should be aware that dialysis duration is likely to increase over time, and should be willing to cooperate with residual function measurements [59].

Incremental dialysis is entirely consistent with the concepts of adequate dialysis dose established in the NCDS and HEMO studies as discussed in Section 1, but incorporates the contribution of residual function, so that dialysis and residual function are seen as both contributing to overall clearance. There are a number of different methods for quantifying combined kidney and dialysis urea clearance (summarised in Appendix 2) which can help with schedule and dose selection. These should be interpreted in clinical context, with due observation of indirect measures of dialysis adequacy such as control of symptoms, blood pressure, fluid gains and electrolytes, so that dialysis dose can be appropriately escalated if treatment appears clinically inadequate.

#### Guideline 2.3 - Conservative schedules

We suggest that lower haemodialysis dose targets may be optimal when quality of life is the primary goal of treatment, rather than longevity. [2D]

#### Rationale

Whilst concepts of dialysis dose have been developed over the last two decades, the dialysis population has been changing, with the median age of the prevalent dialysis population increasing by nearly 20 years, and diabetes becoming one of the leading causes of established kidney failure. For many patients, dialysis is a long-term maintenance therapy that continues until death or dialysis withdrawal, with increasing comorbidity and frailty developing during this time [60].

This changing demographic has important implications for the clinical application of dialysis dose. Firstly, studies have typically focused on younger patients (median age 49 in the NCDS study including no diabetics, and mean age 58 in the HEMO study) so that applying their conclusions in a more elderly group is an extrapolation. Secondly, studies are generally more concerned with mortality, and many strategies in dialysis are aimed at preventing future complications, whereas current symptoms and quality of life are often more relevant to the frailer patient. And thirdly, the burden of dialysis often increases with increasing frailty, so that there is a greater trade-off when considering the burden versus the benefit of treatment. In the context of this changing demography, it is reasonable to question whether conventional dialysis dosing and targets remain appropriate for this population [61].

Frailty as a clinical syndrome can be defined when a number of factors are present including: unintentional weight loss, self-reported exhaustion, weakness and low physical activity. The presence of frailty is associated with increasing disability and hospitalisation, and in dialysis patients, with an adverse quality of life irrespective of dialysis modality.

The optimum dialysis for frail patients has only been studied in small cohorts. Some overlap exists between the features of frailty and those of underdialysis, and it could be argued be that more intensive dialysis might better control some aspects such as fluid overload, intradialytic hypotension or sarcopenia, or conversely that nutritional decline might be accelerated by reduced dialysis. Reductions in dialysis quantity should therefore not be misunderstood as a method of improving these aspects of frailty. However, while increasing hours or frequency of dialysis may theoretically overcome some of these problems, patients often perceive the burden of dialysis on their quality of life more than the symptomatic benefit, and dialysis itself may confer specific harms in this group: a retrospective study identified frequent functional deterioration among dependent patients following the initiation of dialysis [62].

In a challenging clinical area with a paucity of outcome data, it therefore seems entirely appropriate to reduce or disregard numeric targets for dialysis dose, instead individualising dialysis according to specific patient goals. Goal-oriented care is an established approach in patients with multiple co-morbidities which overcomes the problems inherent in disease-specific care processes, with discussions instead concentrating on a patient’s individual aims of treatment.

Shared discussions about dialysis schedule, driven by patient-centred goals can ensure that patients are neither under or over-treated, and in some cases might be a precursor to dialysis withdrawal. Such discussions may need frequent review following changes in the patient’s clinical or personal circumstances.

#### Guideline 2.4 - Paediatric schedules

In children and adolescents we recommend an approach to the assessment of dialysis adequacy which goes beyond biochemical targets, incorporating clinical goals such as growth, bone health, cardiac function and quality of life. [1C]

We recommend targeting dialysis dose to achieve a minimum eKt/V of 1.2 for thrice weekly patients, or a standardized Kt/V of 2.2 for those on augmented schedules. [1C]

We suggest an augmented schedule for children on predominantly liquid nutrition, and those with ventricular systolic dysfunction. [2D]

We recommend a blood flow rate of 5-7ml/kg/min for the majority of patients, using consumables appropriate to body size, with extracorporeal volume less than 10% of the patient’s blood volume. [1C]

#### Rationale

The low incidence of dialysis-requiring kidney disease in childhood, means that many treatment decisions are informed by observational data and studies carried out in adults. The small-solute dose target for adults (eKt/V over 1.2) therefore has some relevance to children, though cautious interpretation of a target extrapolated from a different clinical setting would lead many clinicians to aim for a more conservative (i.e. higher) target dose. In addition, unique physiological aspects of childhood, such as growth, may be improved by increased dialysis dose, and there are strong arguments to suggest that optimum Kt/V may be size-dependent in adults, so that a higher minimum Kt/V may be appropriate [63]. The desirable lower limit for eKt/V is therefore thought to be between 1.2 and 1.4.

However, as is increasingly recognized now in adults, it has long been argued that the optimal quantity of dialysis for children cannot be characterized by a single numerical measurement [64]. In addition to the desirable clinical outcomes shared with adults, the therapeutic goals for children and adolescents receiving dialysis include achievement of normal growth, bone maturation and social development, along with avoidance of cardiac compromise and disrupted education. The increasing evidence that dialysis dose and schedule is able to improve cardiac function and outcomes in many of these domains argues for a broader concept of “adequacy” which might best be assessed using a constellation of clinical outcomes, as well as biochemical targets.

Augmented dialysis, with increased frequency in particular, is therefore increasingly advised by clinicians, and despite the obvious drawback of treatment burden, does not seem to reduce quality of life, even in adolescents [65]. It is possible that augmented schedules are optimal for all children, but some groups seem particularly likely to benefit, including those with cardiac dysfunction and those on a liquid diet, in whom it might otherwise be difficult to achieve safe fluid control [66].

Safe limits appropriate to body size are advocated for many aspects of the extracorporeal circuit, such as a blood flow rate of 5-7ml/min/kg, which is often adequate to achieve dialysis dose with double needles, with arterial aspiration pressures below 200mmHg, to limit endothelial trauma. For single-needle dialysis the highest blood flow rate is obtained using a double pump system (venous flow higher than arterial) monitored by pressure (time pressure regulation), with clamp techniques used to achieve an acceptable compromise between recirculation and blood flow [67]. Consumables appropriate to body size should be selected so that total extracorporeal volume is less than 10% of blood volume, to reduce the volume load with wash-back at the end of the session. System priming with albumin or even blood may sometimes be required for babies and small infants.

#### Guideline 2.5 -Schedules during pregnancy

We recommend counselling women of reproductive age who are receiving or anticipating dialysis, so that they are aware of the interactions between renal replacement therapies and pregnancy which may impact on family planning and modality decisions. [1D]

For dialysis patients wishing to continue their pregnancy, we recommend changing as early as possible to an individualised, augmented haemodialysis schedule. For those with minimal residual function this should be at least 20 hours per week, delivered over at least 6 sessions. [1B]

We recommend an individualised dialysate prescription appropriate to the dialysis schedule and biochemistry results, anticipating the frequent need for a high potassium / low bicarbonate dialysate, supplemented with phosphate. [1C]

We suggest an individualised fluid management protocol, with low ultrafiltration rates and regular clinical assessment, anticipating the typical change in weight during pregnancy. [2C]

#### Rationale

Successful pregnancies in women on haemodialysis are becoming more common: prior to 1995 data from the USA suggested only 40% infant survival, but outcomes in the current era are substantially better [68]. However, pregnancy complications in haemodialysis patients are still more common than in pre-dialysis and transplant patients, and may result in HLA sensitisation, so delaying until after transplantation may be favourable for some. Conception may be more likely with augmented dialysis schedules [69], but the possibility of pregnancy or need for contraception should be considered regardless of dialysis schedule.

The literature linking haemodialysis prescription to outcome in pregnant dialysis patients is limited to case series and systematic reviews [70]. In a recent meta-analysis of 681 pregnancies in 647 patients between 2000 and 2014, authors found that longer weekly dialysis duration significantly associated with a lower incidence of preterm delivery and babies small for their gestational age [71]. More frequent dialysis was also associated with fewer small babies. Normalisation of biochemistry and fluid status appears to give the best outcome, and virtually every publication advocates intensified dialysis.

The best evidence for this approach to date is the comparison of data from the Toronto and US registries of pregnancy in dialysis patients [72]. In women established on dialysis before becoming pregnant, 11 of 13 pregnancies were successful with at least 36 hours per week, compared to 22 of 46 with up to 20 hours (p=0.02). More intensive dialysis was also associated with reduced preterm delivery and greater birth weight.

Residual kidney function facilitates normalisation of fluid and electrolytes, so the accelerated loss of residual function seen with augmented schedules raises concerns about this approach in women with good urine output [73]. In the Toronto/US registry study all of the 17 women who started dialysis partway through the pregnancy had a successful outcome. Since 13 of them were in the shorter treatment group, it appears that longer treatment times would have been unnecessarily burdensome, and possibly detrimental, to patients with significant residual function. In a comprehensive review Hladunewich suggested titrating the dialysis dose to achieve urea 10-15mmol/L after the longest break between sessions [74]. The authors also provide advice on adjusting medication, anaemia management and fetal monitoring which are outside the scope of this guideline.

With augmented schedules dialysate should be individualised, with high potassium / low buffer often required. To ensure the needs of foetal skeletal development are met, low serum calcium and phosphate should be avoided, which may involve adjustment of diet, medication and dialysate: supplementation of the dialysate with phosphate is often necessary. Magnesium should possibly be monitored in the third trimester, since low levels may induce uterine contraction.

Augmented schedules allow patients with minimal residual kidney function to remain close to their target weight and avoid high ultrafiltration rates. Fluid status needs to be assessed frequently during pregnancy, as there is a high risk of fluid depletion, especially in the second and third trimester, and bioimpedance and urine volume may be useful measurements in this setting. Typical weight gains during a healthy pregnancy range from around 150g/week during the first trimester, to around 450g/week during the third trimester.

### Membrane flux and haemodiafiltration

We recommend that patients with minimal residual function should be treated with high-flux dialysers. [1B]

We suggest that haemodiafiltration may be considered as a treatment for intra-dialytic hypotension refractory to other measures, and for dialysis patients with favourable prognosis who are unable or unlikely to be transplanted. [2B]

#### Rationale

##### Convective clearance

Haemodialysis removes uraemic toxins by two very different physical processes: diffusion and convection.

Diffusion is the movement of solutes independent of solvent when the concentration differs between the two sides of a membrane. The rate is dependent on the concentration difference, the diffusion coefficient of the membrane, as well as the blood and dialysate flows, and this process is extremely efficient for small solutes, such as urea. Convection is the movement of those solutes not excluded by pore size, along with their solvent as it crosses the membrane. The rate depends on molecular size and the ultrafiltration rate, and this process is most important for molecules too large for efficient diffusion, but still smaller than the membrane pores, often termed ‘middle molecules’ [18].

Convective clearance is therefore a measurable component of dialysis, which is qualitatively and quantitatively distinct from urea clearance and treatment time. Diffusion of a solute is usually quantified by its Kt/V (Appendix 1) whereas convection is best quantified by its sieving coefficient and the ultrafiltration rate (Appendix 3).

Ascending quantities of convection are therefore achieved with low-flux dialysis, high flux dialysis, and haemodiafiltration. Historically low-flux dialysis was standard, in part because it requires less accurate ultrafiltration control from the dialysis machine - ultrafiltration in standard low-flux dialysis is simply equal to the fluid removed from the patient, usually around 2 litres. In high-flux dialysis pore size is increased, increasing the sieving coefficient for middle molecules, but also the permeability to water is improved, so that internal filtration (bidirectional movement of water within the dialyser) becomes significant: net ultrafiltration of course remains the same, but total ultrafiltration, all of which contributes to middle molecule clearance, is greater and may be as much as 10 litres [75]. In haemodiafiltration a large volume of replacement fluid is given, to allow net ultrafiltration to be increased to around 20 litres (Appendix 3). There is little difference in clearance of small solutes between these methods [76].

Of the (over 100) uraemic toxins known, many are middle molecules (with molecular weight in the range 0.6-60kDa) for which clearance is largely dependent therefore on convection [18]. Convective quantity does not improve clearance of all poorly-diffusing molecules, with phosphate clearance for example, largely unaffected, but clearance of many, such as β-2-microglobulin, is progressively increased by high-flux dialysis and haemodiafiltration [77–79]. A contribution of convective dialysis quantity to favourable outcome is strongly suspected.

##### Membrane flux

Several interventional studies give insight into the impact of membrane flux on dialysis outcomes, for example in the other part of its 2x2 design, the HEMO study group compared high-flux with low-flux dialysis [80]. In the whole group (N=1846) high-flux dialysis did not confer a clear survival advantage (RR 0.92, 95%CI 0.81-1.04) although cardiac mortality was reduced (RR 0.80, 95%CI 0.65-0.99). In the roughly one-third (N=577) of patients with over 3.7 years dialysis vintage prior to randomisation, high-flux dialysis improved survival substantially (RR 0.68, 95%CI 0.53-0.86).

The Membrane Permeability Outcome study randomised 738 incident patients to high vs low-flux dialysis, stratified by serum albumin (normal vs subnormal) [81]. Over a mean observation of 3 years, high-flux dialysis reduced mortality in the low albumin group (N=493, HR 0.63, 95%CI 0.45-0.90) with a less clear reduction in mortality in the whole group. High-flux was similarly advantageous in the subgroup with diabetes.

A meta-analysis of 33 studies comparing high-flux with low-flux dialysis in 3820 patients, found reduced cardiovascular mortality (RR 0.83, 95%CI 0.70-0.99) and a less clear reduction in all-cause mortality (RR 0.95, 96%CI 0.87-1.04) [82]. Endotoxin tends to be absorbed within high-flux membranes, rather than passing through, and initial concerns that dialysate endotoxin would be more problematic with high-flux dialysis appear to have been unfounded [83].

Whilst no study has unequivocally demonstrated the superiority of high-flux dialysis for survival, there is clear evidence of improved cardiovascular outcomes, and all-cause mortality appears to be improved in several subgroups [84]. At the same time, evidence of harm is lacking, all modern machines have accurate ultrafiltration control, and membrane costs are now equivalent. Further research on this question therefore does not seem to be a high priority.

##### Haemodiafiltration

The effect of haemodiafiltration has been informed by four randomised controlled studies, summarised in the table below. In three of these, marginal but non-significant advantages were seen in the haemodiafiltration group, with subgroup analysis suggesting favourable outcome with the highest convection volumes, though the latter to some extent may reflect body size or treatment tolerance [85–87].

A clear advantage with haemodiafiltration was seen in the ESHOL study, which specified a higher convection volume of 23 litres, but consequently may be confounded by subjects’ ability to sustain these volumes, which is dependent on high blood flow, so that censoring may be most frequent in the highest risk patients [88]. Apart from the CONTRAST study [85] these were all analysed ‘as treated’, with right-censoring when treatment was discontinued for any reason, leading to potential bias since endpoints are more likely to be hidden in the haemodiafiltration arm. Another criticism concerns the mechanism of the clinical benefit, since middle molecule levels were not demonstrably improved by haemodiafiltration: plasma levels of β-2-microglobulin increased significantly in both arms of the ESHOL study.

Reduced mortality with haemodiafiltration was observed with pooled analysis of the four studies (HR 0.86, 95%CI 0.75-0.99) due in particular, to a reduction in cardiovascular events, with authors estimating the prevention of one cardiovascular death for every 75 patient-years of treatment [89]. However, due to biases within study designs, considerable doubt over the superiority of haemodiafiltration remains [90].

**Table:** 

Study name (location) Year of main publication	Number Int vs Control Mean age	Interventions Mean observation	Mortality % Int vs Control HR (95%CI) if significant
CONTRAST (Europe/Canada) 2012	358 vs 356 64.1	HDF vs low flux 3 years	36.6 vs 38.8
Turkish HDF (Turkey) 2013	391 vs 391 56.5	HDF vs high flux 1.9 years	13.3 vs 16.6
ESHOL (Spain) 2013	456 vs 450 65.4	HDF vs 92% high flux 1.9 years	18.6 vs 22.8 0.70 (0.53 - 0.92)
FRENCHIE (France) 2017	190 vs 191 76.2	HDF vs high flux 2 years	18.9 vs 22.5

Haemodiafiltration was also assessed in a DOPPS study, in which after adjustment, no association between convection volume and survival was observed [91]. Several of these studies also found a lower frequency of intradialytic hypotension with haemodiafiltration compared to the control group, though the authors acknowledge the difficulty in excluding confounding factors such as cooling and positive sodium balance [92].

### Fluid in haemodialysis (Guidelines 4.1 – 4.2)

#### Guideline 4.1 - Fluid assessment and management in adults

We recommend assessment of fluid status when prompted by clinical circumstances, and on a quarterly basis for stable patients. [1C]

We suggest a multidisciplinary approach to fluid assessment, with patient involvement and the adoption of patient-friendly terminology such as “target weight”, “fluid gain” and “over-hydration”. [2D]

We suggest supplementing clinical assessment of fluid status with a validated objective measurement, such as bioimpedance, at regular intervals, when clinical assessment is unclear, and following an intercurrent illness. [2C]

We recommend a dialysate temperature not greater than 36'C if standardised. [1C]

We recommend avoiding excessive ultrafiltration rates by addressing fluid gains, accepting staged achievement of target weight, or using an augmented schedule, as necessary. [1B]

We recommend prompt nursing intervention to restore haemodynamic stability in symptomatic / severe intradialytic hypotension, with such interventions leading to clinical review. [1C]

#### Rationale

Fluid control is an essential clinical goal of maintenance haemodialysis, but correct fluid management requires clinicians to steer between the two competing / overlapping problems of fluid overload and intra-dialytic hypotension.

Failure to control fluid overload may lead to obvious short-term effects including hypertension and breathlessness, and nephrology trainees quickly become familiar with the emergency dialysis admission with pulmonary oedema. In the longer term also, chronic fluid overload is one of the main drivers of hypertension and is independently associated with poor outcomes: for example, in a US study of over 10 000 prevalent haemodialysis patients, Flythe reported clinical outcomes over 2 years’ follow-up, according to achievement of target weight during the baseline month [93]. Compared to those achieving within 2kg of target weight on at least 70% of sessions, the 15% of patients frequently remaining over-hydrated post dialysis had increased mortality (HR 1.28, 95%CI 1.15-1.43) as did the 7% of patients who were frequently under-hydrated post dialysis (HR 1.22, 95%CI 1.05-1.40).

Often competing, though sometimes associated with fluid control, is intra-dialytic hypotension, which also has immediate consequences familiar in the dialysis unit, including dizziness and cramps, as well as more long-term adverse effects. For example, Sands studied the occurrence of intra-dialytic hypotension (defined as a drop in systolic blood pressure of at least 30mmHg, to below 90mmHg) in 1137 patients in 13 dialysis facilities, over an average period of 3 months [94]. With this definition, hypotension complicated 17.2% of sessions, affecting 74.9% of patients at least once, and 16.2% of patients on at least one third of their sessions. Those most prone to intra-dialytic hypotension were older, more comorbid and with lower pre-dialysis blood pressure, with associated sessional factors including high ultrafiltration volume and non-achievement of target weight. Outcomes associated with intra-dialytic hypotension included shortened survival and increased hospital admission.

The two main treatment parameters by which clinicians aim to optimise fluid control, are target weight and ultrafiltration rate.

Since the earliest days of dialysis, setting ultrafiltration to achieve a set target weight post dialysis, at which the patient is at their correct volume (or “dry”) has been the accepted method of maintaining a consistent volume state, but the method is dependent on accurate estimation of the correct target weight. Though most often assessed by clinical examination, the inaccuracy of this method is widely appreciated so that both overestimation and underestimation are common, with the former contributing to hypertension and left ventricular hypertrophy, and the latter accelerating the loss of residual kidney function and perhaps risking myocardial stunning.

To improve on clinical assessment, nephrologists at one time advocated “probing” target weight: gradual reduction until patients report symptoms suggesting hypovolaemia, but this may reduce treatment compliance and a more collaborative approach is more common: where possible, patients should be asked to participate in monitoring their fluid status. To this end terminology should be simple and intuitively understood: for example, when discussing target weight, the term “dry weight” can give the impression that the aim is to remove as much fluid as possible, and “ideal weight” can be confusing as it is also used to describe the preferred body mass index. Although less accurate, “hydration” is a more familiar term than “volume” as a description of fluid status. Stable patients should be assessed for target weight changes perhaps quarterly, but staff and patients should be particularly vigilant when changes in flesh weight are likely, such as following hospital admission, or when starting nutritional supplementation. Fluid management often requires input from a multidisciplinary team, so a documented policy may ensure that the approach is consistent.

Improvement on clinical assessment using objective methods for selecting target weight has been sought for a long time, though no single measurement has so far gained widespread acceptance. Methods have fallen into one of a number of categories: imaging (such as inferior vena cava diameter), biochemistry (such as brain natriuretic peptide), electrophysiology (such as bioimpedance) and dynamic intradialytic measurement (such as blood volume monitoring). Many publications address one or more of these methods, and several detailed reviews are available.

Some of these studies suffer from the limitations of self-referencing design (demonstrating that the use of method X to guide selection of target weight, reduces the frequency of over-hydration as defined by method X) and improvement in clinical outcomes are often harder to demonstrate. For example, Leung studied intradialytic hypotension in 32 haemodialysis patients during 8 weeks of standard care and 8 weeks during which ultrafiltration was informed by blood volume monitoring, but no advantage was seen in terms of hypotension frequency or symptoms [95].

No clear recommendation can be made regarding the optimal method, but when clinical assessment feels uncertain, it seems very reasonable to supplement this with an objective measure, and bioimpedance has some of the most promising data on clinically relevant endpoints. In a randomised study of 156 patients, Nur used bioimpedance data to adjust target weight in the intervention group, whilst control patients had bioimpedance measured but not available to treating physicians [96]. Over the 12 month study, bioimpedance-defined fluid overload was reduced in the intervention group, as was blood pressure and left ventricular mass index (131±36 to 116±29g/m^2^, p<0.001).

Regardless of the final volume achieved, the rate of ultrafiltration appears separately to influence intra-dialytic hypotension and clinical outcome. In a DOPPS study of 22 000 patients in 7 countries, Saran observed that an ultrafiltration rate over 10ml/h/kg was associated with both intra-dialytic hypotension (RR 1.30, p=0.04) and mortality (RR 1.09, p=0.02) [97]. And using data from the HEMO study (N = 1846) Flythe divided patients according to ultrafiltration rate into three groups: less than 10, 10-13, and over 13ml/h/kg, demonstrating increased mortality in the highest ultrafiltration rate group (HR 1.59, 95%CI 1.29-1.96) [98]. In the same study, when treating ultrafiltration rate as a continuous variable (using a cubic spline method) the authors identified 10ml/h/kg as the threshold beyond which mortality begins to increase, possibly quite sharply.

These studies are non-interventional, therefore associations are with observed (rather than prescribed) ultrafiltration rate, and there is also a close interaction with session length (since rate is obviously the volume over the time) but these data provide a convincing argument for avoidance of excessive rates. This should not however be at the expense of non-achievement of target weight and acceptance of over-hydration (though staged achievement over a number of sessions is frequently appropriate) but rather should focus clinicians on session length or addressing fluid gains between dialysis sessions. The ultrafiltration required during dialysis depends on the degree of over-hydration present at the start of the session, so restricting fluid intake reduces ultrafiltration rate, and is part of standard advice for the majority of patients. Consideration must be given to the cause of increased fluid intake such as habitual drinking or thirst associated with either dietary sodium intake or raised blood glucose. Advice on managing fluid intake is therefore best delivered on an individualised basis, as part of a dietary management plan to support adherence and patient experience. This topic is covered in guidelines for the nutritional management of kidney disease.

Other relevant aspects of the dialysis prescription include dialysate sodium and temperature.

Sodium balance, thirst and fluid control are also influenced by dialysate sodium. Many observational studies report lower fluid gains and lower blood pressure in patients treated with low dialysate Na (typically 136-138mmol/l). Antihypertensive treatment is frequently overlooked in large studies, but reasonable supportive evidence can also be found in interventional studies. For example, Gumrukcuoglu reduced dialysate sodium from 140 to 137mmol/l in 41 patients over 6 months, reporting reduced fluid gains, and no blood pressure change but a reduction in antihypertensive use from 1.9 to 1.2 agents per patient [99]. This potential benefit was not without drawbacks however: in common with other groups, investigators also found that cramps and intra-dialytic hypotension became more frequent.

Lowering dialysate sodium therefore does appear to improve fluid control and blood pressure, albeit with some side effects, however another note of caution arises from observations on mortality in different dialysate sodium groups. Studying almost 30 000 patients from DOPPS phases 1-4, with dialysate sodium varying between 138 and 142mmol/l in 90% of patients, Hecking found that higher dialysate sodium was, as expected, associated with modestly increased fluid gain and systolic blood pressure (increasing by 0.17% body weight and 0.66mmHg per 2mmol/l increase in dialysate sodium) [100]. However, when addressing indication bias by studying only the 56% of facilities using a standardised dialysate sodium, they found that higher dialysate sodium was unexpectedly associated with reduced mortality (HR 0.88 per 2mmol/l increase in dialysate sodium, 95%CI 0.83-0.94). There is insufficient consistency in the literature for a clear recommendation on dialysate sodium, though if a standardised dialysate sodium is used for all patients, some clinicians would avoid a choice below 140mmol/l.

Dialysate temperature has been consistently associated with intra-dialytic hypotension. Even thermoneutral haemodialysis (temperature-matched so that the dialysis circuit neither heats nor cools the patient) leads to an increase in core temperature, though it is not clear if this is due to reduced heat loss (for example due to cutaneous vasoconstriction) or increased thermogenesis (for example due to increased cardiac output) [101]. Reduced dialysate temperature has therefore been the subject of a number of interventional studies and two meta-analyses [102, 103].

In the most recent of these, Mustafa reported on 26 studies totalling 484 patients [103], observing an average 70 (95%CI 49 - 89) percent reduction in hypotension, though with an increase in cold-related symptoms. Twenty-four of these studies however were either small (less than 20 patients) or of short duration (less than 3 sessions). The two largest studies provide further insight: in Maggiore's study of 95 patients over 12 sessions

[104], isothermic (in which dialysate temperature is set so that core temperature is unchanged) rather than thermoneutral dialysis reduced hypotension from 50 to 25% of sessions. In Fine’s study of 128 patients over 10 sessions [105], 35'C dialysate rather than 37'C similarly reduced hypotension, but the benefit was seen only in those with subnormal temperature before dialysis. Preventing temperature rise therefore appears to be more important than cooling, which may be achieved on an individual basis using dialysate 0.5 - 1.0 degree lower than core temperature or in the whole unit by using dialysate temperature 36'C or lower. The latter is probably adequate for most patients, with individualisation seeming a reasonable option for those with persisting hypotension or cold-related symptoms, and it is reasonably clear that if a standardised dialysate temperature is being used, then the choice should be at or under 36'C.

Regardless of the quality of dialysis prescription, intra-dialytic hypotension will still occur, in some patients more than others, for which prompt nursing intervention is essential [106]. Common measures include leg raised positioning, ceasing ultrafiltration, and fluid administration (saline being as good as albumin and far cheaper [107]). Measures for “simple” intra-dialytic hypotension should be coupled with assessment for underlying intercurrent illness (such as infection or cardiac arrhythmia) or less commonly a specific dialysis complication (such as air embolism or dialyser reaction). Frequent intervention should lead to re-assessment of target weight / ultrafiltration setting and a medication review - in some cases predialysis hypertension may be preferable to dialysis intolerance. Specific pharmacological measures are rarely used but the alfa-agonist Midodrine has reasonable supportive evidence: in meta-analysis the average improvement (increase) in systolic/diastolic post-dialysis blood pressure was 12.4/7.7mmHg [108].

#### Guideline 4.2 - Paediatric fluid considerations

In growing children we recommend clinical assessment of fluid status and target weight, and dietetic assessment, at least monthly. [1C]

We suggest supplementing clinical assessment with a validated objective measure of fluid status such as bioimpedance, on a monthly basis or more frequently during periods of rapid growth or illness. [2C]

We recommend regular assessment of ultrafiltration tolerance, using extended times to avoid excessive ultrafiltration rates. [1D]

#### Rationale

Assessment of target weight in children and adolescents is particularly challenging as it needs frequent adjustment in line with growth or periods of illness. This is particularly true for infants and adolescents during rapid phases of growth. Overestimation of target weight will result in chronic fluid overload leading to hypertension and left ventricular hypertrophy, whereas chronic under-hydration is likely to detrimentally affect residual kidney function and lead to increased symptomatic hypotension both during and immediately post-dialysis. Hypotensive tendency is also multifactorial and cannot alone be relied on to ascertain a patient’s target weight. It is therefore essential that target weight is adjusted at least on a monthly basis following clinical assessment, in conjunction with dietetic review [109, 110].

### Dialysate (Guidelines 5.1 – 5.4)

When the 2^nd^ edition of the RA Guidelines was published in 1997, the only recommendation relating to the composition of the dialysate was that renal units phase out the use of acetate in favour of bicarbonate buffering, since the improved efficiency of dialysis could overwhelm the capacity to metabolise acetate. The need to keep bicarbonate separate from divalent cations to prevent precipitation meant that dialysate had to be produced using two different concentrates, leading to the modern proportioning system in which sodium bicarbonate is mixed with an electrolyte concentrate (‘acid concentrate’) at the point of use, allowing independent control of most dialysate constituents. Some dialysate constituents have diversified whereas others have gradually become standardized.

Dialysate calcium was often supra-physiological in the 1990’s (around 1.75mmol/L) to prevent hypocalcaemia, but this became unnecessary with increasing use of vitamin D analogues and calcium-containing phosphate binders, so that dialysate calcium has become reasonably standardized, usually in the range 1.25-1.50mmol/L. Non-standard dialysate calcium may sometimes be helpful, for example in the context of calciphylaxis, but this is usually driven by bone-mineral considerations and is outside the scope of this guideline.

In the 1990’s, dialysate was usually glucose-free due to cost and microbiological concerns, and hypoglycaemia was often a problem for diabetic patients. Glucose containing dialysate was initially prescribed for diabetic patients, but extended to all as costs improved, so that a dialysate glucose of 5.5mmol/L is now standard in almost all UK dialysis units. The other constituent of dialysis that has become standardised is magnesium, with low (usually 0.25 or 0.375mmol/L) or high (usually 0.75mmol/L) magnesium being replaced by a dialysate magnesium of 0.5mmol/L, close to the lower end of the normal range.

Opposing these trends, there has been significant diversification in dialysate potassium, and similarly, buffer concentrations and practices vary between units and manufacturers, and are discussed below.

#### Guideline 5.1 - Selection of dialysate potassium

We recommend an optimal pre-dialysis serum potassium in the range 4.0–6.0mmol/L, remembering to consider measurement errors (e.g. due to haemolysis) when interpreting levels. [1B]

We suggest choosing dialysate potassium between 1.0 and 3.0mmol/L for the majority of patients, using an individualised approach, in general using the highest dialysate potassium that is sufficient to control pre-dialysis hyperkalaemia. [2C]

We suggest a combined approach to managing hyperkalaemia, which may include decreasing dialysate potassium and/or other measures, including dietary advice, medication review and increased dialysis frequency. [2D]

#### Rationale

Historically, it was often difficult to remove the potassium accumulated between dialysis sessions, so dialysate potassium between zero and 2mmol/L was common. The requirement for dialysate with potassium levels that are close to, or within, the normal range reflects the increased efficiency of modern dialysis and the increased age of the modern patient. In most units dialysate potassium is determined by the choice of acid concentrate: zero potassium is no longer used, and suppliers offer concentrates with potassium between 1 and 4mmol/L.

Removal of accumulated potassium by intermittent haemodialysis inevitably leads to a fluctuating profile of serum potassium with a risk of cardiac arrhythmias at both high and low concentrations. This probably contributes to the clustering of sudden cardiac death around the peridialytic period, and at the end of the weekend gap [111].

Both low and high pre-dialysis potassium are associated with increased mortality, so that the mortality curve is U-shaped. Low potassium often appears more harmful in unadjusted data: for example, in a study of 483 Taiwanese patients followed from 2004 to 2008, Hwang showed that those with pre-dialysis potassium below 3.5mmol/L had more than twice the risk of mortality than those with higher levels [112]. But this link may be due to confounding by comorbidity malnutrition: in a much larger study of 74219 patients between 2001 and 2004, a U-shaped risk curve was seen, with increased mortality with pre-dialysis potassium outside the range 4.3–5.6mmol/L [113]. After adjustment for case mix and malnutrition parameters however, the increased risk of mortality remained only for the high potassium patients (though the less than 4.0mmol/L category was not subdivided). The optimum pre-dialysis potassium therefore appears to be above 4.0 with an upper limit between 5.6 and 6.0mmol/L, though the broader range seems more appropriate given the considerations below.

The relationship between post-dialysis potassium and mortality is unknown, as it is rarely measured, but the risks of post-dialysis hypokalaemia can be inferred from studies of dialysate potassium [114, 115]. For example, Pun compared 502 patients who experienced sudden cardiac arrest in dialysis units between 2002 and 2004, with 1632 age and vintage matched controls, finding that risk was doubled if the patient last dialysed with a low dialysate potassium (less than 2.0mmol/L) [163].

The DOPPS review of modifiable practices associated with sudden death included 36235 patients in 12 countries of whom 6606 were dialysed with dialysate potassium at least 3.0mmol/L [116]. An increased risk of sudden death was observed with dialysate potassium below 3.0mmol/L (HR 1.17, 95%CI 1.01–1.37), though it was not clear if this risk extended to those with pre-dialysis serum potassium over 5.0mmol/L. Others have suggested that lower dialysate potassium may prevent sudden death in this subgroup [111, 113], but the latest DOPPS analysis found no meaningful difference in mortality or arrhythmia events between patients treated with dialysate potassium of 2.0 or 3.0mmol/L [117].

The understandably strong impulse to control pre-dialysis hyperkalaemia should therefore be tempered by consideration of the less visible risk of post-dialysis hypokalaemia. Pragmatically therefore one can conclude the following general principles:

Firstly, pre-dialysis hyperkalaemia should be controlled, though an overly tight range may be counterproductive, so the previously recommended target for pre-dialysis potassium still seems optimal (4.0 – 6.0mmol/L). Caveats to interpreting this range should be noted: firstly, achievement of pre-dialysis potassium within this range does not necessarily mean that dialysate potassium is optimal, and secondly, consistent adherence to treatment is most likely just as important as specifics of the potassium range or dialysis prescription.

Secondly, non-dialysate approaches to hyperkalaemia may sometimes be more favourable [118, 119]. Dietary reduction may be preferable if it can be achieved without an adverse effect on protein-calorie intake, and other dialysis changes may be appropriate, such as increasing blood flow, duration or frequency. Consideration could also be given to potassium binding resins [120].

Thirdly, lower dialysate potassium does increase the removal of potassium during each session [121], and based on the risk of arrhythmias due to hyperkalaemia, dialysate potassium should be reduced if other measures are not possible or successful [122]. However, dialysate potassium should be no lower than is necessary to achieve this goal – individualization does therefore seem necessary, so that each patient uses the highest dialysate potassium which still controls pre-dialysis hyperkalaemia. This pragmatic approach has probably driven the steady increase in the use of higher potassium dialysates, and reduction in the use of concentrations below 2.0mmol/L, over the 5 DOPPS phases between 1996 and 2015 [117].

Finally, and particularly for measurements taken remote from the laboratory, the relatively high frequency of measurement errors (for example due to in vitro haemolysis) should be remembered when interpreting potassium levels.

#### Guideline 5.2 - Selection of dialysate buffer

We recommend an optimal pre-dialysis serum bicarbonate in the range 18.0-26.0mmo/L, remembering to consider measurement errors (e.g. due to exposure to air) when interpreting levels. [1C]

We suggest the term ‘dialysate buffer’ rather than ‘dialysate bicarbonate’ to avoid confusion arising from differences in manufacturers’ terminology. [2C]

We suggest choosing dialysate buffer below or equal to 37.0mEq/L for the majority of patients, using a standardised or individualised approach. [2C]

We suggest a combined approach to abnormal pre-dialysis serum bicarbonate, which may include increasing dialysis dose, oral bicarbonate, nutritional support, or individualising dialysate buffer. [2D]

#### Rationale

We suggest a combined approach to abnormal pre-dialysis serum bicarbonate, which may include increasing dialysis dose, oral bicarbonate, nutritional support, or individualising dialysate buffer. [2D]

The literature on dialysate bicarbonate is difficult to interpret due to unclear definitions when reporting the bicarbonate and additional alkali components. Most commonly the electrolyte concentrate contains a non-bicarbonate acid, to reduce the deposition of calcium and magnesium salts – acetic acid is perhaps the most common, but citric acid and sodium diacetate may also be used.

When mixed to form the dialysate, acetate reacts with sodium bicarbonate to form sodium acetate, water and carbon dioxide:

$$ {\mathsf{H}\mathsf{C}}_{\mathsf{2}}{\mathsf{H}}_{\mathsf{3}}{\mathsf{O}}_{\mathsf{2}}+{\mathsf{NaHCO}}_{\mathsf{3}}\to {\mathsf{NaC}}_{\mathsf{2}}{\mathsf{H}}_{\mathsf{3}}{\mathsf{O}}_{\mathsf{2}}+{\mathsf{H}}_{\mathsf{2}}\mathsf{O}+{\mathsf{CO}}_{\mathsf{2}} $$

The addition of 3mmol of acetic acid to a litre solution containing 35mmol of bicarbonate therefore reduces the bicarbonate concentration to 32mmol/L. In publications, bicarbonate concentration in this dialysate may variably be referred to as having a bicarbonate concentration of 32 or 35mmol/L, with the acetate content rarely reported.

In addition, the bicarbonate ‘setting’ on machines from different manufacturers, refers variably to the bicarbonate concentration either prior to (eg. Braun) or after (eg. Fresenius) mixing with the electrolyte concentrate. The terms ‘actual’ bicarbonate (because that is what is actually added as sodium bicarbonate) and ‘final’ bicarbonate (because that is the bicarbonate in the dialysate at the point of use) are sometimes used to separate their meaning. However, the total buffer concentration remains the same before and after this mixing, so this term has a clear unambiguous meaning (equivalent to the sum of bicarbonate and acetate concentrations in the final dialysate). In a discussion of the DOPPS study of dialysate bicarbonate, Tentori observed that when asked either for the bicarbonate or total buffer concentration, most DOPPS units returned the same figure, suggesting that clinicians generally mean ‘actual’ rather than ‘final’ bicarbonate, which is the same as total dialysate buffer [123–125].

The factors affecting pre-dialysis serum bicarbonate levels include protein intake, residual kidney function, interdialytic fluid gain, dialysate buffer concentration, dialysis adequacy, oral sodium bicarbonate and other alkaline medications such as calcium carbonate [126].

Observational studies of pre-dialysis levels usually show a J-shaped mortality curve, with most of the excess risk associated with high levels of bicarbonate [127, 128], but this appears to be due to the close link between high bicarbonate and malnutrition. For example, in a study of 56385 between 2001 and 2003, Wu observed a progressive increase in mortality as pre-dialysis bicarbonate increased beyond 23mmol/L, but also strong associations between higher bicarbonate and worsening markers of nutrition including albumin, phosphate and protein intake [129]. When adjusted for comorbidity and 12 parameters associated with malnutrition, most of the increased mortality appears with low bicarbonate, at levels below 18–21mmol/L. Some guideline groups have therefore increased the lower limit for optimal pre-dialysis bicarbonate to 20 or 22mmol/L [130, 131].

Post-dialysis bicarbonate is rarely measured, but three considerations argue for caution in attempting to achieve a minimum pre-dialysis bicarbonate. Firstly, the risks associated with abnormal bicarbonate are less clear and of a lower magnitude than those associated with abnormal potassium (mortality hazard ratio of approximately 1.2 for the most extreme category of bicarbonate versus 1.5 for potassium).

Secondly, although it is principally low bicarbonate which carries risk, high pre-dialysis bicarbonate also appears to be harmful. Whilst much of the risk observed is attenuated by adjustment, pre-dialysis bicarbonate is still associated with increased mortality at levels above 27mmol/L [129]. Additionally, an increased risk of peri-dialytic cardiac arrest has been observed with high pre-dialysis bicarbonate: a Fresenius Medical Care memo in 2011 reported an internal case-control study of 941 patients in 667 facilities who suffered cardiac arrest in 2010. Risk was 4.7 times higher in patients with pre-dialysis bicarbonate over 28mmol/L, and 6.3 times higher if they also had pre-dialysis potassium below 4mmol/L [132].

Thirdly, high dialysate buffer is associated with increased mortality. For example, in a large study of dialysate buffer using DOPPS data (collected from 17031 dialysis patients in 11 countries between 2002 and 2011) Tentori observed a lower risk of mortality in patients treated with dialysate buffer less than or equal to 32mmol/L, regardless of pre-dialysis bicarbonate (HR 0.90, 95%CI 0.80–1.01) and higher risk with dialysate buffer at or above 38mmol/L (HR 1.07, 95%CI 0.97–1.19) [123].

Pragmatically therefore one can conclude the following general principles:

Firstly, pre-dialysis acidaemia should be controlled, though an overly tight range may be counterproductive, so the previously recommended lower target for pre-dialysis bicarbonate still seems optimal, though the upper target could safely be increased (18.0–26.0mmol/L). As with potassium, achievement of this range does not necessarily ensure optimal dialysis prescription.

Secondly, dialysate buffer at or over 38mmol/L should generally be avoided, and the optimal dialysate buffer for the majority of patients is probably in the region of 32–35mmol/L.

Thirdly, many other factors affect pre-dialysis bicarbonate, the dominant ones being nutritional state and dialysis dose, so that abnormalities of pre-dialysis bicarbonate should not lead clinicians automatically to think of adjusting dialysate buffer. High bicarbonate in particular should prompt a nutritional thought process initially. It is not clear that adjustment of dialysate buffer is a helpful strategy for optimising pre-dialysis bicarbonate, or that such an adjustment has much impact on pre-dialysis bicarbonate levels. Specific groups however, such as patients with abnormal levels despite optimal diet and dialysis strategy, may have something to gain from dialysate buffer adjustment. Conversely, increased dialysate buffer may be more hazardous in certain circumstances, such as in combination with low potassium dialysate [122, 133].

Whilst it is a very reasonable thing to do, and might prove to be beneficial in future studies, it is not currently clear that individualization of dialysate buffer is superior to standardization.

Finally, and particularly for measurements taken remote from the laboratory, the relatively high frequency of measurement errors (for example due to carbon dioxide escape) should be remembered when interpreting bicarbonate levels [134, 135].

#### Guideline 5.3 - Supplementation of dialysate with phosphate

We suggest considering supplementation of the dialysate with phosphate in patients on augmented dialysis schedules. [2D]

#### Rationale

The conventional haemodialysis patient struggles to achieve sufficient phosphate removal, and historically dialysate has always been phosphate-free. Guidelines usually focus more on the upper limit than the lower limit for optimal pre-dialysis phosphate and ranges in the region of 1.1-1.7mmol/L are often suggested, with most of the emphasis on treatments to reduce phosphate - indeed, most of the Renal Association's advice on phosphate can be found in the guideline on mineral-bone management. However, with demographic and

treatment trends of the last decade, low phosphate is becoming more common, and since the symptoms of hypophosphataemia are non-specific [136], this problem may be easily overlooked.

The relationship between pre-dialysis phosphate and mortality is J-shaped, with increased risk occurring at both high and low levels. But phosphate is strongly associated with age and nutritional state, so that the mortality risk associated with low phosphate is substantially (although incompletely) attenuated by adjustment for comorbidity and malnutrition [137]. In the context of low pre-dialysis phosphate therefore, the main clinical focus should be on nutritional assessment and support.

When patients are unable to consume sufficient phosphate to match intradialytic loss, supplementation of the dialysate is a logical approach to managing hypophosphataemia. The argument for supplementation is generally accepted in the context of augmented dialysis, when post-dialysis phosphate is often measured, and may be found to be very low in well-nourished patients [138]. It is common practice, for example, to supplement dialysate with phosphate in pregnant patients receiving daily dialysis.

Supplementation could also be used to prevent undesired loss of phosphate in patients on conventional regimes with low pre-dialysis phosphate that is refractory to other measures [139]. While this does appear to be clinically helpful in case reports, data to support this approach remain limited. However, as patients with low pre-dialysis phosphate currently receive a form of dialysis which inevitably worsens this abnormality, so the instinct to ‘do no harm’ may be a sufficiently persuasive argument for some clinicians.

Phosphate precipitates in solutions containing calcium or magnesium, so like bicarbonate, must be added to the electrolyte concentrate at the point of use, but there is currently no commercially available phosphate additive approved for use in intermittent haemodialysis [140, 141]. ‘In house’ supplementation can be achieved by adding phosphate salts to the electrolyte concentrate at the start of the session, but solutions intended for intravenous use typically contain potassium and are too dilute. Pharmaceutical grade phosphate salts in powder form can be used, but require quality assurance on storing, weighing, adding and ensuring complete dissolution. The most common method is therefore ‘off label’ use of solutions intended as enemas: Cleen (formerly Fleet) Enema for example, is very suitable for enriching dialysate [142], although it contains antimicrobial preservatives (benzalkonium chloride and disodium edetate) which are widely used in medical products such as eye drops, which might have adverse effects in this context. The use of Cleen Enema in dialysate has a good safety record however: Pierratos first reported its use in nocturnal dialysis in the late 1990s [143], and frequent dialysis programmes in many countries have adopted this method [144, 145]. Practical advice on adding phosphate to dialysate is provided in Appendix 4.

#### Guideline 5.4 - Paediatric dialysate considerations

We recommend individualisation of dialysate electrolyte concentrations, including potassium, buffer and calcium. [1C]

We suggest an individualised dialysate temperature, between core temperature and 0.5°C below, with monitoring of intradialytic core temperature for neonates and smaller children. [2D]

#### Rationale

Adult guidelines for dialysate composition (sections 5.1 – 5.3) are generally applicable to children, though there are a number of additional considerations.

In children with residual kidney function, tubular dysfunction is not uncommon, leading to electrolyte wasting and hypokalaemia or acidosis. Calcium balance is also more complex in children: the normal range for calcium is age-dependent and growing children require a positive calcium balance, so that hypocalcaemia may be both more common and more harmful, and yet vascular calcification is sometimes seen even in children and adolescents, in whom calcium-phosphate product is an important risk factor [146, 147]. Similarly, dietary protein intake is often proportionately greater than that of adults, and pre-dialysis acidosis therefore more common. The complexity and clinical heterogeneity of these issues therefore argues strongly for a more individualized approach to dialysate composition in children [148].

Thermal exchanges during dialysis may also be more significant particularly in neonates and younger children, due to the proportionately greater blood flow, and sometimes a reduced capacity for compensation due to body size. Hypothermia should therefore be avoided by individualising dialysate temperature, with intradialytic monitoring in those most at risk. Control of thermal exchanges is available on some modern dialysis machines.

### Anticoagulation

We recommend that patients without increased bleeding risk should be given unfractionated or low-molecular-weight heparin during dialysis to reduce clotting of the extracorporeal system. [1A]

We recommend that systemic anticoagulation should be omitted or minimised in patients with increased bleeding risk. [1C]

We recommend that patients with heparin allergies should be prescribed a non-heparin form of anticoagulation. [1A]

#### Rationale

Platelet activation in the extracorporeal circuit accelerates thrombin generation via the intrinsic coagulation pathway, so that anticoagulation is usually required to prevent thrombosis. Unfractionated heparin is used as the standard anticoagulant worldwide in view of its proven efficacy, ease of use and long safety record unless the patient has recent or active bleeding, thrombocytopenia, heparin allergy or heparin induced thrombocytopenia.

With a mean half-life of 1.5 hours, heparin is usually administered as a loading dose of 1000-2000 IU followed by a continuous infusion of 500-1500U/h that is discontinued approximately 30 minutes before the end of the dialysis session. Monitoring can be performed by measuring the activated partial thromboplastin time ratio (aPTTr) or the whole-blood activated clotting time aiming for around 150% of pre-dialysis or centre normal values [149, 150]. But in practice the bolus dose, infusion rate and stopping times are adjusted empirically, according to clot formation in the dialysis circuit, and the time for needle sites to stop bleeding. Heparin dose may need to be increased with higher haematocrit, or reduced / withdrawn in patients at risk of haemorrhage, those with thrombocytopenia or on long term anticoagulation [151].

Alternatively, a low molecular weight heparin may be used [152], having a longer half-life, given as a single ‘arterial limb’ bolus at the start of dialysis [153]. Although monitoring can be performed using anti-Xa activity, these are not always available and laboratory testing correlates less directly with clinical effect, so as with unfractionated heparin, dose adjustment is usually empirical, but larger or repeated doses may be needed depending on convective clearance and session length, and reduced doses for those at risk of haemorrhage [154]. Several systematic reviews comparing low-molecular-weight with unfractionated heparin have found no difference in the incidence of bleeding complications, post-dialysis access bleeding, or thrombosis of the extracorporeal circuit [155–158]. With its convenience for nursing staff, the use of low-molecular-weight heparin is becoming more common in Europe.

For patients at increased risk of bleeding, several options are used in clinical practice. Firstly, several techniques require no anticoagulation to be administered during dialysis, including: combining a high blood flow rate and regular pre-dialyzer circuit flushing every 15-30 minutes [159, 160]; using a heparin coated dialyzer [161, 162]; adding heparin to the rinsing solution [160]; or using a dialysate containing citrate [163–165].

Secondly, a regional anticoagulant can be used such as citrate, prostacyclin (epoprostenol) or nafamostat (not currently available in UK). Regional anticoagulation with citrate [166] and epoprostenolol [167] have both been reported to reduce the risk of haemorrhage compared to heparin, though there are drawbacks: epoprostenol may induce hypotension and is costly, whereas citrate administration requires re-infusion of calcium based on electrolyte monitoring, adding complexity and nursing staff time [168]. Finally, lower doses of unfractionated or low-molecular-weight heparin have been used with caution in patients at risk of bleeding [151, 154].

Heparin induced thrombocytopenia, usually occurring shortly after regular exposure to heparin, and sometimes with thrombosis, may occur in heparin-treated dialysis patients [169, 170]. The risk of heparin induced thrombocytopenia can be estimated using the 4T scoring system [171], and is usually confirmed by laboratory testing and detailed guidelines on diagnosis and treatment are published by the British Society of Haematology, but in suspected or confirmed cases, all heparins should be withdrawn [172]. The risk of thrombosis increases with the severity of thrombocytopaenia, and anticoagulation is usually started with either the direct thrombin inhibitor argatroban [173], or a natural (danaparoid) or synthetic (fondaparinux) heparinoid [174, 175]. Argatroban is reversible, given by continuous infusion, and requires careful laboratory monitoring with aPTTr. The heparinoids are renally excreted and have prolonged half-lives in dialysis patients, such that monitoring of the bolus given with a dialysis session can be based on anti-Xa activity prior to the following session. Once the platelet count returns to normal, patients are usually anticoagulated with warfarin, but in the majority of cases antibodies disappear with time, and patients have been successfully re-challenged with unfractionated and low-molecular-weight heparins once laboratory testing becomes negative [176].

### Adverse events during dialysis (Guidelines 7.1 – 7.3)

#### Guideline 7.1 - Routine blood loss

We suggest that during washback, dialysis lines and dialyser are observed to ensure residual blood loss is kept to a minimum. [2C]

#### Rationale

A small amount of blood loss occurs during normal haemodialysis, for example due to blood retained in the dialyser and circuit after washback, and bleeding into the dressing over needling sites, but there is no clear consensus as to what constitutes a ‘normal’ quantity of blood loss due to dialysis. The literature on minimising blood loss during haemodialysis is sparse, and much of the evidence is of limited quality.

The weighed gauze method has been to quantify bleeding after removal of needles, with ‘excessive’ defined as blood-soaked gauze weighing over 4g [177]. And excessive bleeding has been associated with poor outcomes, for example in a study of 4152 dialysis sessions in 143 patients, Lin found that excessive bleeding following dialysis needle removal occurred regularly, and was associated with lower haemoglobin levels [178, 179]. Kalantar-Zadeh suggested patients can lose up to 3g iron per year, with one gram being lost in the lines and dialyser, and a further gram lost in blood sampling [180]. Though it is unclear how they are derived, these estimates suggest that up to 20ml per session may be normal.

In a comparison of buttonhole versus rope-ladder cannulation in 33 patients, Verhallen found no difference in bleeding times after needle removal between the two techniques [181]. Various suggestions have been made, for example McCann suggested needling at an angle of 25 degrees [182], and Fruits suggested flushing the arterial dialysis needle with saline, and reducing the amount of blood drawn for testing, but none of these measures is well supported by clinical evidence [183]. Currently there is insufficient evidence therefore to support any recommendations regarding blood preservation and management of vascular access.

Clotting of the dialysis circuit leads to much greater blood loss than is routine. Adequate but safe anticoagulation is an important component of prevention, and is covered elsewhere in this guideline, but regular monitoring during dialysis and observation of the colour of the lines and dialyser post-dialysis, also play a role. This concept is supported in literature, for example Kalocheritis noted the contribution of this type of blood loss to anaemia, and the relevance of human factors [184]. Reasonable consensus therefore supports the importance of nursing observation, particularly during washback.

No evidence was found regarding the effects of excessive blood sampling on blood loss. Daugirdas and Tattersall point out that on-line measurement of adequacy may reduce the need for blood sampling, but describe the benefits mainly in respect of cost and staff time [185]. However, ensuring that blood samples are taken only when required for routine monitoring or for additional diagnostic indications, is perhaps obvious common sense.

#### Guideline 7.2 - Disconnection haemorrhage

We recommend maintaining awareness of the risk of disconnection, the limitations of pressure alarms, and importance of direct observation, through a program of education, including patients and carers. [1D]

We suggest regular assessment of individual risk, so that high risk patients can have enhanced monitoring, which could include specific devices. [2B]

#### Rationale

Disconnection leading to haemorrhage may occur at any part of the dialysis circuit, though venous needle dislodgement may be the most frequent and serious, with rapid blood loss occuring at the rate of the blood flow pump, until it is detected. Disconnection incidents are thought to be uncommon, but the true prevalence is uncertain due to inconsistent reporting. Once detected, management begins with haemostasis and fluid resuscitation, as with any major haemorrhage, and the literature concentrates instead on methods to minimise risk and enhance detection, with publications available from the EDTNA/ERCA and the American Nephrology Nurses Association [186, 187].

Variability in human processes is recognised as an important factor, and most units have established protocols to ensure consistency in aspects of care such as taping needles in position to minimise the chance of disconnection [188].

Dialysis machines have several types of safety monitor [189] and if disconnection does occur, the drop in pressure should be detected and cause the machine to alarm. However, it has been repeatedly demonstrated that these alarms cannot be relied on to detect all cases [190]. The use of asymmetric windows (such as -30 to +70mmHg) may be helpful to maximise the detection of disconnection, while minimising alarms from increases in pressure at the venous needle [191, 192].

Because machine alarms cannot be relied on, direct observation remains important, involving vigilance on the part of nursing staff, and unit management, so that lines of sight are not obscured, patients are not dialysing alone and their vascular access sites are not covered. Because of the low prevalence of disconnection, complacency may develop: continuous education is therefore advocated to ensure awareness amongst healthcare staff, patients and their carers [193].

Risk of disconnection is greater in some patients, and enhanced monitoring may be appropriate based on individual risk assessment. Simply placing patients closer to the nursing desk may be sufficient, but reliable monitoring can also be achieved by use of blood loss detection devices, which typically are secured at the site of vascular access and alarm on the detection of blood [194, 195]. Device monitoring may be appropriate for patients at high risk, such as confused or agitated patients, and may have a greater role in home haemodialysis programmes [196–199]. One interventional study considered the effect of blood loss detection devices on nursing staff, showing an improvement in self-reported feeling of safety when devices were used [200].

#### Guideline 7.3 - Immune reactions during dialysis

We recommend that dialysis staff should be aware of the features and management of dialysis reactions, and should have access to a range of dialyser types. [1C]

#### Rationale

From the early 1980s reports appeared describing abrupt clinical reactions occurring soon after the onset of dialysis [201]. These have traditionally been classified into two types.

Type A reactions were said to affect less than 1% of patients per year, often re-occurring in the same patient, with onset within the first few minutes of dialysis. Mainly occurring with first use, rather than re-used dialysers the features were quite ‘anaphylactic’ in nature (itching, flushing, bronchospasm, hypotension, sometimes with burning at the access site) and often severe, with cardiac arrest occasionally described [202]. Associated with eosinophilia, these reactions were caused mainly by residual ethylene dioxide (used to sterilize membranes) with antibodies detectable in many cases [203]. Similar reactions were described to polyacrylonitrile membranes, especially in ACE inhibitor treated patients (by increasing kinin activation) and in hydrogen peroxide treated re-used membranes [204]. Immediate cessation of dialysis was usually necessary, along with anaphylaxis-type treatment. Extra rinsing or a change of membrane sterilisation would often prevent reoccurrence.

Type B reactions, said to be more common, occurring later in the dialysis session, were typically less severe, improving with continued dialysis. Characterised mainly by chest and back pain (also sometimes with vomiting, breathlessness and hypotension) they were caused by complement activation and pulmonary cell sequestration, and associated with transient reductions in circulating white cells. These reactions were clearly linked with the ‘bio-incompatibility’ of cellulose-based membranes [205].

**Fig. 2:**
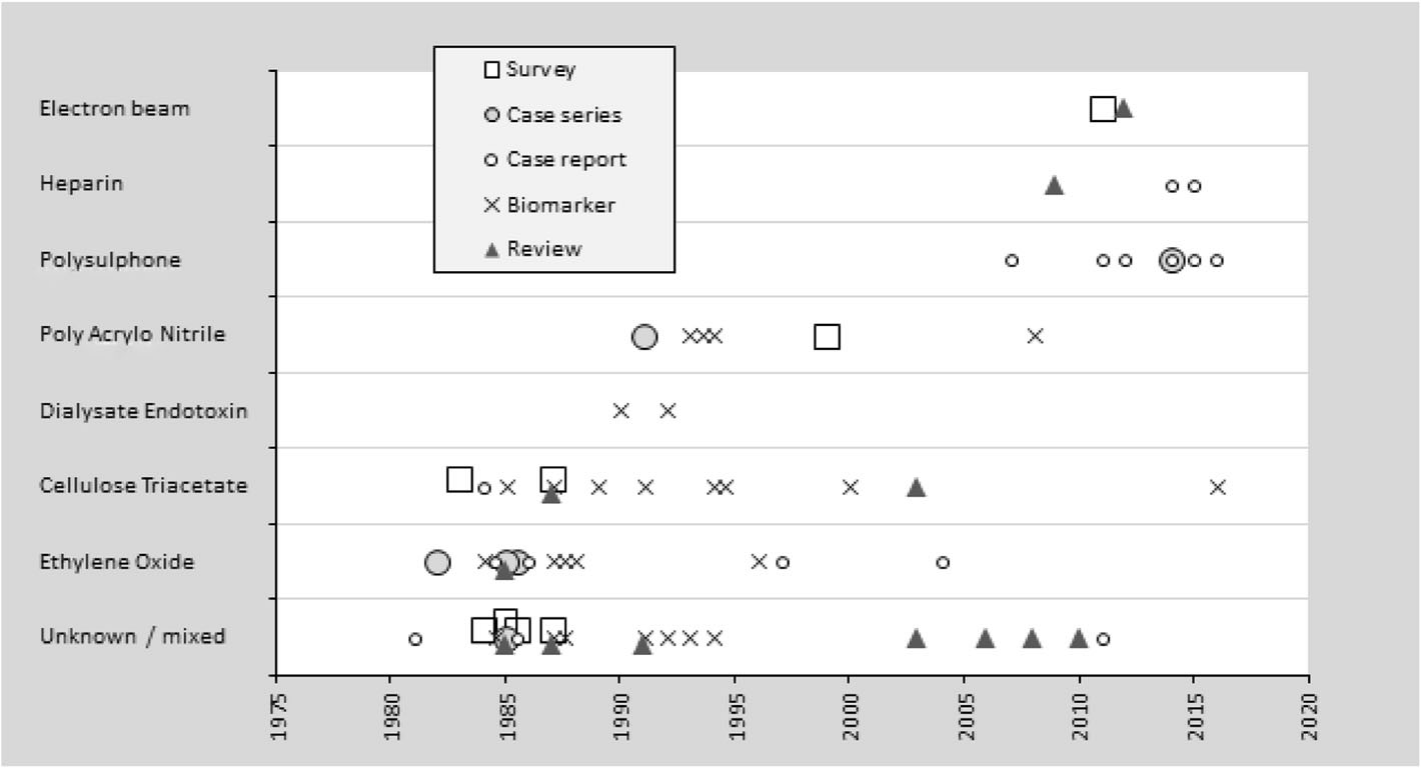
Literature timeline showing the changing epidemiology of dialysis reactions

Dialyser re-use, ethylene dioxide sterilisation and unmodified cellulose membranes are all now very uncommon, and as dialysis practices have evolved, the epidemiology of these reactions has changed, reflected in the changing literature (Fig. 2). In modern practice dialysis reactions are uncommon but do still occur, including polysulphone allergy, heparin allergy and isolated thrombocytopenia.

Reactions with ‘type A’ (anaphylactic) features continue to occur with polysulphone membranes, though many variants are described, including those with fever as the predominant symptom [206]. Eosinophilia is an important clue, though not invariably present, and other blood tests (tryptase, total IgE) may be useful [207]. The diagnostic hallmark is resolution of the syndrome following a change of membrane type, and (though little guidance is available from literature) anaphylaxis treatments are often given, with steroid pre-treatment sometimes used before dialysis sessions. Stopping ACE inhibitors may also reduce the severity.

Reactions to intra-dialytic heparin are sometimes described, ranging in severity from asymptomatic to a serotonin-like syndrome of breathlessness and flushing, often with hypertension. These are usually but not always associated with thrombocytopenia (persisting between dialysis sessions) and thrombotic complications may occur. Transient asymptomatic thrombocytopenia has also been described, often recovering between dialysis sessions so that pre-dialysis platelet count may be normal. This reaction has been associated with electron beam membrane sterilization, but the mechanism is unknown [208].

Several complications other than dialyser reactions may present with similar peri-dialytic symptoms. More common ones include bacteraemia and hypovolaemia, whilst disequilibrium, air embolism and the chloramine / hard water syndromes are rarer. Water purification complications may be more common in the home haemodialysis setting.

### Patient experience of dialysis (Guidelines 8.1 – 8.4)

#### Guideline 8.1 - Home haemodialysis

We recommend that home haemodialysis should be available in all units as part of a comprehensive renal replacement therapy programme. [1A]

We suggest training patients and/or care partners to achieve a defined set of competencies, using an individualised approach to training method and speed. [2D]

We suggest units form a contract with patients outlining responsibilities, including an agreement to dialyse as per prescription and trained technique, and including a policy for re-imbursement of directly arising patient costs. [2D]

We suggest supporting patients with a specific team including nephrologists, technicians, and nurses, with rapid access to dialysis in-centre when required. [2C]

We suggest an agreed individualised prescription for home haemodialysis, taking into account lifestyle goals, with the same dose and time target considerations as centre-based patients. [2C]

We recommend enhanced safety measures for patients who dialyse alone or overnight, and an enhanced risk assessment for patients with blood-borne viruses. [1C]

#### Rationale

There is increasing evidence of the benefits of augmented haemodialysis schedules, in terms of both outcome and health-related quality of life, but providing more frequent dialysis in-centre is a challenge in the UK, and it is widely recognised that augmented schedules are most easily accommodated in the home setting [33, 35, 209–212]. The literature on home haemodialysis and augmented schedules therefore overlaps substantially, but home

haemodialysis additionally is increasingly acknowledged to provide a level of convenience and flexibility not achievable in-centre.

Despite these benefits the penetration of home haemodialysis in the UK remains low, comprising only 0.4% of incident and 2% of prevalent dialysis patients. Many organisations such as NICE and KDIGO promote universal availability for clinically suitable patients, acknowledging that collaborative working between centres maybe required [213, 214]. But it is clear from registry data that variability of access still exists, with some centres not offering this modality, and considerable variation in uptake between centres.

Home haemodialysis patients must be able to manage their dialysis safely, and monitor their condition. Modality decisions should be supported by a full assessment of clinical and social circumstances, as well as the home environment, including a discussion of the impact of therapy on others within the household [215]. It is essential that patient and carer expectations and fears are appropriately addressed before commencing training [217]. Few data are available to guidance on clinical suitability, but the ability to complete training may be more important than clinical diagnosis: a number of programmes have reported that patients with complex comorbidities can improve with more frequent therapy, more tailored to their needs [222, 223].

Training on a ‘1 to 1’ basis with a specific training staff is widely accepted as optimal, with the learning style and training duration adapted to the individual [221]. Type of vascular access should not be a limiting factor, but appropriate training, surveillance and technique assessment form essential parts of the home haemodialysis programme [224, 225].

The success of a home haemodialysis programme is dependent upon a skilled and specific multi-disciplinary team facilitating education, training and patient support in the community, and optimal individual outcomes are dependent on patient understanding, and appropriate cooperative liaison with this support [218]. This may be facilitated with an explicit contract, so that the manner in which this clinical responsibility is shared is clear. The financial responsibility for treatment rests with the provider, and re-imbursement of directly arising patient costs should be readily available [216].

A home haemodialysis Programme requires adequate medical, nursing and technical support, and should support at least 12 to 20 patients, and train at least 10 patients per year in order to maintain appropriate staff expertise and cost effectiveness, so smaller renal units may find it more appropriate to share resources with other centres. Minimum safe staff to patient ratios are not well defined, but recommendations for peritoneal dialysis (such as minimum of 1 nurse per 20 patients) may be relevant [218–220]. However, as training for home haemodialysis is more complex, additional staffing should be considered to ensure that training new patients does not detract from the support of established patients [217]. Patient mix should also be considered, so that programmes with a greater number of complex patients are staffed more favourably [224, 226].

Home haemodialysis patients should receive the same level of medical supervision, and the same monitoring and dose considerations as in-centre patients, and as for other patients, the schedule should be individualised depending on patient values and therapeutic goals. Dialysis dose should be quantified as for other augmented schedules, but should be interpreted with the flexibility of the patient’s schedule also in mind. To ensure that the home dialysis team can provide the best possible support that is responsive to the individual, recording of sessional details by the patient or carer is desirable [229].

Specific circumstances may require additional risk assessments and/or additional measures: enhanced safety measures, for example to detect disconnection, should be available for patients dialysing alone or overnight, and protection of household contacts of patients with blood-borne viruses should be considered, particularly for those directly involved in therapy [187, 224, 227, 228].

#### Guideline 8.2 - Shared haemodialysis care

We suggest that all centre-based haemodialysis patients should have opportunity and encouragement to learn aspects of their dialysis treatment, and take an active role in their care. [2D]

#### Rationale

There is little research that has been directly conducted into shared haemodialysis care, however there is considerable evidence of the benefits of supported self-care in other long term conditions [230]. Low health literacy amongst dialysis patients is associated with worse survival [231] whereas self-motivation and education can result in better care, for example, in phosphate control and fluid balance [232, 233]. As with the broader NHS, dialysis services are experiencing considerable pressure to deliver high quality in the face of fiscal challenge, and an important mechanism to ensure that quality of care is maintained, is to engage service users as true partners in their own treatment: self-management is an ambition in ‘Kidney Health: Delivering Excellence’ [234]. To achieve this, health care professionals need to enhance their roles, becoming educators and facilitators, supporting patients to take a greater role in their own care, and increasing their opportunities for dialysing at home.

Shared haemodialysis care impacts on all domains of health. Central among these are: the enhanced patient safety that comes from education on infection control (see the WHO campaign ‘Save lives: clean your hands’ [235]); the enhanced equity consequent on offering all patients training in their treatment rather than only those planning haemodialysis at home; and the enhanced experience when patients can put themselves onto dialysis, or manage their own alarms, without waiting for a nurse [236].

The process of haemodialysis can be broken down into approximately 14 tasks (Appendix 5). The exact arrangements may vary between units but the concept is essentially the same: that centre-based patients are given the opportunity to train to perform one or more of these tasks. It is key that patient involvement is voluntary, and that learning is individualised to the style and speed of the individual. Shared haemodialysis care is associated with a range of barriers and enablers that are best explored through quality improvement work, in order to design favourable conditions for successful implementation.

#### Guideline 8.3 - Intradialytic exercise

We recommend that intradialytic exercise should be available in all units, as a treatment for enhancing physical functioning, in patients without contraindications. [1B]

We suggest that intradialytic exercise be considered as a method of enhancing quality of life. [2C]

We suggest that exercise regimes be devised by appropriately trained staff. [2C]

#### Rationale

Whilst cardiovascular disease remains the principal causes of death in dialysis patients [237], there is a significant interaction with body composition, with muscle wasting in particular exacerbating mortality [238]. Muscle wasting and poor physical fitness also reduce functional abilities including activities of daily living, thus reducing quality of life in haemodialysis patients [254]. However, muscle wasting is modifiable by exercise, and epidemiological studies suggest that regular exercise can even reduce mortality [239], but unfortunately daily physical activity is typically low in haemodialysis patients, perhaps due to the time burden and symptoms associated with treatment [240].

Based on evidence from eight systematic reviews and meta-analyses [241–248], analysing data from 1000 adult participants on dialysis, the clinical effectiveness of exercise on physical function and health related quality of life can be summarised as follows:

1. Despite the high-risk status of dialysis patients, no serious exercise-related adverse events have been reported from over 30 000 patient-hours of exercise observed [244, 246]. Adverse events reported include post-exercise hypotension, fatigue, myalgias, painful feet, and aggravation of foot ulcers, though not with increased incidence in exercise groups. Compliance with exercise programmes ranged from 43 to 100%, and dropout rates from 15 to 50%.
2. Short term (2-6 months) prescribed exercise of any type, frequency and intensity, resulted in significant and clinically moderate/large improvement in cardiorespiratory fitness, with a mean increase in peak VO2 of 5ml/kg/min [243, 246].
3. Any prescribed exercise delivered during hemodialysis sessions produced significant and clinically moderate improvement in muscle strength [245], with a mean increase of 9.9kg [243].
4. Any type of prescribed exercise consistently produced significant and clinically large improvements in some indices of functional capacity, such as ‘sit to stand’ transfers [247], whereas other indices, such as walking performance, were improved according to some reviews [247] but not others [243, 245].
5. Self-reported physical function was significantly improved in exercising patients [247]. This often contributes to quality of life scores, and may therefore explain why some studies conclude that exercise improved quality of life.

Taken together there is therefore good evidence that the uptake of regular exercise improves physical function and quality of life in haemodialysis patients, without causing significant harm, and that delivery of exercise within haemodialysis sessions can achieve this.

Exercise during the dialysis process may also assist with solute clearance. Enhanced urea clearance is predicted by modelling but an impact on Kt/V is found in some studies (nine of eighteen studies reviewed) but not others [249], whereas improvements in phosphate clearance and serum levels are consistently observed [249–253].

Some evidence suggests the type of exercise most likely to be beneficial: larger improvements were observed with interventions delivering a progressively increasing exercise volume, at least three times per week, for at least 30 minutes, lasting for at least four months, and including an additional resistance-training component [244, 246–248]. Comparative evidence for specific exercise programmes is currently unavailable, but some guidance on practical implementation of intradialytic exercise is offered in Appendix 6.

#### Guideline 8.4 - Dialysis experience for children and adolescents

We recommend that haemodialysis for children and adolescents should be delivered in a dedicated paediatric dialysis centre or at home, with the involvement of a paediatric multidisciplinary team. [1C]

We recommend that adolescents should commence an active transition programme by 14 years, or at the time of presentation in those already over 14. [1D]

#### Rationale

Haemodialysis sessions are associated with physical symptoms, social restriction, and loss of control, which for children and adolescents may be particularly depersonalising and unpleasant. These effects may be mitigated by an appropriate environment and trained support staff, and in-centre dialysis is therefore best delivered in a dedicated unit, with paediatric nephrologists working alongside the full multidisciplinary team, including nurses, dietitians, psychologists, play therapists, teachers and social workers [148, 255, 256]. In this way children can be supported to reach their full potential despite the burdens of treatment. The first dialysis session is of particular importance in establishing therapeutic trust and parental confidence - psychological preparation for this event can alleviate anxiety, reduce symptoms and improve the tolerability of dialysis.

Children and adolescents can be supported to take on aspects of their own care, often along with parents or guardians, and are likely to gain as much benefit as adults from involvement in a shared care program [257]. And home haemodialysis has many advantages for children, allowing an augmented schedule without institutionalisation, and providing a flexibility which can reduce the impact of dialysis on social development.

Transition describes the process of preparing adolescents, along with their families, for the move from paediatric to adult care. It should be individualised, taking into consideration the physical and psychological development of the adolescent, and requires a variable amount of time [258]. Adolescents will suffer the least disruption if moved to adult care following engagement with a transition programme, and should be introduced to the concept of transition in early adolescence (12-14 years). For those over 14 when presenting to paediatric services, transition planning should commence immediately alongside other aspects of care.

## Data Availability

Not applicable

## Appendix 1

### Simplified mathematics of urea clearance

**Urea Kinetic Modelling and Kt/V**

In the design of the NCDS study, ‘dose’ of dialysis was defined as a target for time-averaged urea (low urea = high dose group). However, urea level in dialysed patients depends as much on protein intake as the amount of dialysis: loss of appetite may lead to low urea, so this cannot be relied on to indicate sufficient dialysis. The concept of Kt/V emerged from this study, where K is dialyser clearance (of urea), t is treatment time and V is volume of distribution (of urea). Despite its appearance, Kt/V is not intended as something to calculate from its constituents K, t and V, since only t is accurately known, but by consideration of the following relationship: the rate of removal of urea is proportional to its concentration. Following this basic rule, the concentration of urea after a dialysis session of duration t, is therefore approximated by:

$$ {\mathsf{U}}_{\mathsf{post}}={\mathsf{U}}_{\mathsf{pre}}\ \mathsf{x}\ \mathsf{e}\hat{\mkern6mu} \left(-\mathsf{Kt}/\mathsf{V}\right) $$

where

$$ {\mathsf{U}}_{\mathsf{post}}=\mathsf{urea}\ \mathsf{post}\ \mathsf{dialysis} $$

$$ {\mathsf{U}}_{\mathsf{pre}}=\mathsf{urea}\ \mathsf{pre}\ \mathsf{dialysis} $$

Kt/V is therefore the negative exponent in a numerical model describing the fall in urea during a single dialysis session. Different forms of Kt/V reflect models with differing levels of complexity, which in addition to the concept above, may also take account of other aspects such as: urea removal by convection, urea generation, and separate “pools” within which urea is distributed.

The gold standard Kt/V is derived from ‘Urea Kinetic Modelling’: iterative computation to give the best fitting coefficients for urea clearance and generation, based on three urea measurements (spanning an interdialytic interval and a dialysis session). This approach is broadly accepted, but although online programs are available (e.g. Solute Solver, www.ureakinetics.org) the non-formula method hinders widespread use, and a number of simplified approaches are more common - three are commonly used and summarised here.

**Log-Ratio and Urea Reduction Ratio**

If the basic model above is used, ignoring other factors, with urea assumed to be distributed evenly in body water, then Kt/V is given by the log ratio:

$$ \mathsf{Kt}/\mathsf{V}=-{\mathsf{\log}}_{\mathsf{e}}\ \left[\ {\mathsf{U}}_{\mathsf{post}}/{\mathsf{U}}_{\mathsf{pre}}\ \right] $$

This simple form of Kt/V, sometimes called the “Log-Ratio”, is mathematically related to the Urea Reduction Ratio (URR), the reduction in urea as a ratio of the pre-dialysis level, often expressed as a percentage:

$$ \mathsf{URR}\ \mathsf{100}\ \mathsf{x}\ \left({\mathsf{U}}_{\mathsf{pre}}-{\mathsf{U}}_{\mathsf{post}}\right)/{\mathsf{U}}_{\mathsf{pre}} $$

$$ \mathsf{Kt}/\mathsf{V}={\mathsf{\log}}_{\mathsf{e}}\ \left[\ \mathsf{100}/\left(\mathsf{100}-\mathsf{URR}\right)\ \right] $$

A threshold for URR is therefore precisely the same as a threshold for Kt/V calculated by the Log-Ratio.

URR is the simplest measure of dialysis dose, and is the current audit measure collected by the Renal Registry. URR has the disadvantage that it does not account for clearance due to ultrafiltration, urea generation or urea rebound. It tends to over-estimate dialysis dose in shorter treatments, and under-estimate dose in treatments longer than 4 hours. However, it is long established and there is no argument over its calculation.

**Single-pool Kt/V (spKt/V)**

Although the above is technically also a single-pool method, the term ‘single pool Kt/V’ usually refers to the method proposed by Daugirdas, which calculates Kt/V by a formula taking account of the effect of ultrafiltration:

$$ \mathsf{spKt}/\mathsf{V}=-{\mathsf{\log}}_{\mathsf{e}}\ \left[\ \left({\mathsf{U}}_{\mathsf{post}}/{\mathsf{U}}_{\mathsf{pre}}\right)-\left(\mathsf{t}/\mathsf{7500}\right)\ \right]+\left[\ \left(\mathsf{UF}/\mathsf{TW}\right)\ast \right(\mathsf{4}-\left(\mathsf{3.5}\ast \left({\mathsf{U}}_{\mathsf{post}}/{\mathsf{U}}_{\mathsf{pre}}\right)\right)\ \Big] $$

where

$$ \mathsf{t}=\mathsf{dialysis}\ \mathsf{t}\mathsf{ime}\ \left(\mathsf{\min}\right) $$

$$ \mathsf{UF}=\mathsf{ultrafiltration}\ \left(\mathsf{litres}\right) $$

$$ \mathsf{TW}=\mathsf{target}\ \mathsf{weight}\ \left(\mathsf{kg}\right) $$

This method takes account of more factors but consistently over-estimates Kt/V. Targets have therefore often been expressed with an adjustment factor if using spKt/V (eg. “eKt/V = 1.2, or spKt/V = 1.4”).

**Equilibrated Kt/V (eKt/V)**

The dynamic interaction between urea concentrations in intra- and extra-cellular pools, results in reduced effectiveness of treatment because dialysis removes urea only from the extracellular pool, which is then refilled from the intracellular pool during and after dialysis. This results in a gradual rebound in urea concentration for approximately one hour after dialysis, so that if post-dialysis urea is measured immediately, then the treatment effect is over-estimated.

Equilibrated Kt/V attempts to correct for this by using a post-rebound urea measurement:

$$ \mathsf{eKt}/\mathsf{V}=-{\mathsf{\log}}_{\mathsf{e}}\ \left[\ {\mathsf{U}}_{\mathsf{post}-\mathsf{rebound}}/{\mathsf{U}}_{\mathsf{pre}}\ \right] $$

Due to the inconvenience of measuring urea post-rebound (an hour after dialysis has finished) a formula is used to adjust for this reasonably predictable effect using urea measured immediately. More than one equation has been published but there is very little difference between them. This one is known as the Tattersall version:

$$ \mathsf{eKt}/\mathsf{V}=\left(\mathsf{spKt}/\mathsf{V}\ast \mathsf{t}\right)/\left(\mathsf{t}+\mathsf{35}\right) $$

Apparent urea rebound is dependent on vascular access, so 35 is replaced by 22 in patients dialysing via a catheter. This method is non-biased and has most often been used in studies of dialysis dose, such as the HEMO study. Targets given in terms of other measures of dialysis dose such as URR may be useful in comparing units, but are not accurate for individual patients.

All forms of Kt/V (including eKt/V) implicitly use V to normalise for body size, whereas body surface area is conventionally used to normalise renal function. There are theoretical reasons to believe that normalisation of Kt/V to other body size parameters such as body surface area would be more useful, but these concepts have not gained widespread acceptance.

The Log-Ratio is a reasonable approximation of eKt/V since the underestimation (from ignoring ultrafiltration and urea generation) is offset by overestimation (from ignoring urea rebound). In fact, for a 4 hour session, with UF/TW = 0.02, in a fistula patient, the Log-Ratio is almost identical to eKt/V, with URR = 70 being equivalent to eKt/V = 1.2. However, in most other settings, Log-Ratio will underestimate or overestimate dose, so that the target URR required to achieve eKt/V = 1.2 needs to be varied, as in this table:

**Table:**

Urea Reduction Ratio equivalent to eKt/V = 1.2				
Time (h) by access type		UF/TW		
Fistula	Catheter	0.02	0.03	0.04
3.5		71.0	70.2	69.4
4.0		70.0	69.2	68.4
4.5	3.5	69.1	68.3	67.5
5.0	4.0	68.2	67.4	66.6
	4.5	67.4	66.6	65.8
	5.0	66.8	66.0	65.2

## Appendix 2

### Adjusting dialysis for residual function

Since residual renal clearance (Kru) is continuous, and dialysis clearance is intermittent (with Kt/V referring to clearance during a single dialysis session), the quantities of both cannot simply be added. When it is planned to reduce dialysis dose by incorporating the contribution of residual function, it is necessary to somehow add these clearances so that the dialysis component is appropriate. Three methods of combining these clearances have been proposed.

**Converting Kru to an equivalent eKt/V (Combined eKt/V)**

In this method, residual kidney function is converted to an equivalent per-session eKt/V, by multiplying by a factor F, which empirically inflates the time over which residual clearance is measured, to account for its greater efficiency compared to that of dialysis, with the value of F depending on dialysis frequency. A “Combined Kt/V” is calculated:

$$ \mathsf{Combined}\ \mathsf{eKt}/\mathsf{V}=\mathsf{eKt}/{\mathsf{V}}_{\mathsf{Dialysis}}+\mathsf{eKt}/{\mathsf{V}}_{\mathsf{Kidney}} $$

$$ \mathsf{eKt}/{\mathsf{V}}_{\mathsf{Kidney}}=\mathsf{Kru}\ast \mathsf{F}/\mathsf{Vu} $$

where:

$$ \mathsf{eKt}/{\mathsf{V}}_{\mathsf{Dialysis}}\ \mathsf{is}\ \mathsf{calculated}\ \mathsf{as}\ \mathsf{above} $$

$$ \mathsf{Vu}=\mathsf{volume}\ \mathsf{of}\ \mathsf{distribution}\ \mathsf{of}\ \mathsf{urea}\ \left(\mathsf{ml}\right),\mathsf{approximated}\ \mathsf{by}\ \mathsf{580}\ast \mathsf{TW}\ \left(\mathsf{kg}\right) $$

$$ \mathsf{F}=\mathsf{5500}\ \left(\mathsf{for}\ \mathsf{thrice}\ \mathsf{weekly}\ \mathsf{schedules}\right) $$

With the method above, a Combined eKt/V can be calculated and the dialysis component adjusted to achieve a target Combined eKt/V of 1.2.

**Converting Kt/V to an equivalent renal clearance (EKRc)**

An alternative method is to convert per-session Kt/V into an equivalent continuous renal clearance and then add it to Kru. Casino and Lopez computed kinetic estimates of combined dialysis and kidney urea clearance (normalized to volume) which they termed “equivalent renal urea clearance” (EKRc). In the absence of residual function, an eKt/V target of 1.2 equates to EKRc 13ml/min. For a thrice weekly schedule EKRc is given by the formula:

$$ \mathsf{EKRc}\ \left(\mathsf{ml}/\mathsf{\min}\right)=\mathsf{1}+\left(\ \mathsf{10}\ast \mathsf{eKt}/\mathsf{V}\ \right) $$

With this method Kru is added to EKRc and the dialysis component adjusted to achieve a total of 13ml/min.

**Converting both eKt/V and Kru to a weekly dialysis dose (stdKt/V)**

Dialysis schedule and dose can be converted to an equivalent weekly clearance, “Standard Kt/V” (stdKt/V) proposed by Gotch, based on kinetic models which relate urea generation to average weekly pre-dialysis urea. This allows comparison between schedules: a frequent schedule with stdKt/V = 2.1 is equivalent (in terms of small solute clearance) to a thrice-weekly schedule with eKt/V = 1.2. Residual function can be incorporated into stdKt/V (sometimes termed “Total Standard Kt/V”) by formulas available [259].

## Appendix 3

### Quantifying convection

Convective clearance of a toxin depends firstly on the sieving coefficient. Seiving coefficient (SC) is a measure of the ease with which a solute passes through the membrane relative to solvent, so this depends largely on the relative size of the solute molecule and the membrane pore cross-section. It can be measured during pure ultrafiltration as the ratio of solute concentration before and after passing through the membrane. SC takes values between 0 (the molecule is unable to pass through the membrane) and 1 (the molecule passes just as easily as water).

Secondly, convective clearance depends on the ultrafiltration rate.

In pure haemofiltration, clearance is achieved entirely by ultrafiltration. The loss of fluid by ultrafiltration is replaced by infusion into the blood downstream of the filter (“post-dilution”). In this case, clearance (K) for any solute is equal to convective clearance (Kc) and can be calculated from:

$$ \mathsf{K}=\mathsf{Kc}=\mathsf{Quf}\ast \mathsf{SC} $$

where:

$$ \mathsf{Quf}=\mathsf{ultrafiltrate}\ \mathsf{flow}\ \mathsf{rate} $$

In haemodiafiltration (HDF), there is a mixture of diffusion and convection. Diffusion reduces the concentrations in the ultrafiltrate, reducing Kc. Clearance in haemodiafiltration can be predicted by the following equation:

$$ \mathsf{Kc}=\mathsf{Quf}\ast \mathsf{SC}\ast \left(\ \left(\mathsf{Qp}-\mathsf{Kd}\right)/\mathsf{Qp}\ \right) $$

$$ \mathsf{K}=\mathsf{Kc}+\mathsf{Kd} $$

where:

$$ \mathsf{Qp}=\mathsf{plasma}\ \mathsf{flow}\ \mathsf{rate} $$

$$ \mathsf{Kd}=\mathsf{diffusive}\ \mathsf{clearance} $$

The effect of this is to diminish the convective component. In post-dilution HDF, convection will always increase total clearance, but this increase is negligible for small solutes which diffuse easily (such as urea). For larger molecules (such as beta-2-microglobulin) convection can significantly and usefully increase clearance.

Pre-dilution HDF, in which replacement fluid is given upstream of the dialyser, is less commonly practiced, and more complex mathematically. Convection is similarly reduced by diffusion as above, but in addition both convection and diffusion are substantially reduced by the dilutional effect of the replacement fluid. The ultrafiltrate rate and volume are usually adjusted to account for this.

In publications the convective component of HDF is quantified as the filtration volume. This means the total sessional filtration volume in post-dilution HDF, or the equivalent adjusted volume in pre-dilution HDF.

## Appendix 4

### Adding phosphate to dialysate

Cleen Ready-To-Use enema comes in a 133ml pack costing 68p (March 2017). The pack is designed to deliver a 118ml dose containing 21.4g of sodium acid phosphate (sodium dihydrogen phosphate dihydrate NaH_2_PO_4_.2H_2_O) and 9.4g sodium phosphate (disodium phosphate dodecahydrate Na_2_HPO_4_.12H_2_O). This gives a concentration of 1.38mmol/ml phosphate.

The acid concentrate, together with the added enema, will be diluted according to the proportioning ratio of the dialysis machine. The volume of enema (V_E_) that needs to be added to the canister is given by:

$$ {\mathsf{V}}_{\mathsf{E}}\ \left(\mathsf{ml}\right)={\mathsf{C}}_{\mathsf{PO4}}\ast {\mathsf{V}}_{\mathsf{AC}}\ast \mathsf{DF}/\mathsf{1.38} $$

where:

$$ {\mathsf{C}}_{\mathsf{PO4}}=\mathsf{target}\ \mathsf{dialysate}\ \mathsf{phosphate}\ \left(\mathsf{0}.\mathsf{4mmol}/\mathsf{l}\ \mathsf{is}\ \mathsf{commonly}\ \mathsf{used}\right) $$

$$ {\mathsf{V}}_{\mathsf{AC}}=\mathsf{volume}\ \mathsf{of}\ \mathsf{the}\ \mathsf{acid}\ \mathsf{concentrate}\ \left(\mathsf{e}.\mathsf{g}.\mathsf{6L}\right) $$

$$ \mathsf{DF}=\mathsf{dilution}\ \mathsf{factor}\ \mathsf{for}\ \mathsf{the}\ \mathsf{dialysate}\ \left(\mathsf{eg}.\mathsf{35}\ \mathsf{for}\ \mathsf{machine}\ \mathsf{proportioning}\ \mathsf{at}\ \mathsf{1}:\mathsf{34}\right) $$

Using these numbers, for example, gives an enema volume of 61ml to be added to the concentrate. Note that this formula depends on the formulation of the enema and is specific to Cleen.

This simple formula ignores the small change in concentrate volume, as the effect is below the 5% tolerance allowed for electrolyte concentrations for haemodialysis and related therapies (ISO 23500 Part 4). Albalate used the above formula to prepare dialysates with C_PO4_ between 0.48 to 1.12mmol/l, reporting good agreement between measured and target C_PO4_ using Gambro and Fresenius machines and a range of acid concentrates [260].

Sodium content in the phosphate-enriched acid concentrate will be reduced (since the enema sodium is 37g/l, compared to around 83g/l for an acid concentrate that proportions at 1:34, and 107g/l for one that proportions at 1:44). This will lead to a lower dialysate sodium (by 0.7 to 0.9mmol/l) with volumetric proportioning, and a higher concentrate pump rate with conductivity feedback. It is possible that this effect could trigger machine alarms, requiring machine re-programming.

## Appendix 5

### Haemodialysis tasks for shared care

List of dialysis-related tasks that an individual may choose to learn to perform, as part of shared care:

1. Washing your hands prior to all procedures and arm (as appropriate) if fistula or graft is used.
2. Recording your weight
3. Recording your blood pressure and pulse
4. Recording your temperature
5. Setting up your dialysis machine
6. Preparing your dressing pack
7. Programming your prescription on the dialysis machine
8. Putting your needles in or preparing your access line
9. Connecting your lines and commencing dialysis
10. Responding to alerts from your dialysis machine
11. Disconnecting lines and completing your dialysis
12. Applying pressure to needle sites after dialysis or
13. Locking your own access line
14. Administering any of your injections.

## Appendix 6

### Implementing intradialytic exercise

We suggest the following guidance for implementation of intradialytic exercise:

- Exercise should be supervised for greatest compliance and efficacy by an appropriately trained individual (e.g. physiotherapist, sport scientist, cardiac rehabilitation specialist or an assistant physiotherapist/dietitian/nurse with additional training from one of the former groups).
- Exercise should be completed between 30 min and two hours of the dialysis procedure.
- Exercise should be completed during all dialysis sessions (unless contraindicated).
- Exercise should include both aerobic (e.g. intradialytic cycling) and resistance (e.g. TheraBands and/or lifting of ankle weights) components of lower or upper body, dependent on access site.
- Exercise should be preceded by warm up activities (e.g. exercising for a minimum of five minutes, gradually increasing intensity until one half of prescribed training intensity is obtained).
- Exercise should be completed for at least 30 minutes per hemodialysis session.
- Exercise should be completed at moderate to vigorous intensity, ranging from 40-75% of VO_2_ reserve or heart rate reserve (if a graded exercise test is completed). When a graded exercise test cannot be completed, and for muscle conditioning exercises, a rating of perceived exertion on the Borg CR100 scale of “moderate” (20) to “strong heavy (50)” or on the Borg RPE scale of 12 to 15 can be used *with caution* as a method to prescribe exercise intensity.
- Volume of exercise should be progressed gradually by adjusting duration, frequency, and/or intensity until the desired exercise goal (maintenance) is attained.
- Exercise should be followed by cool down activities (e.g. exercising for a minimum of five minutes, starting at one half of prescribed training intensity and gradually decreasing intensity until exercise is stopped).
- Once patients are established exercising during dialysis, they should be encouraged to complete additional exercise on non-dialysis days.
- To enhance adoption and adherence in novice exercisers, moderate-intensity activity should be prescribed.
- To maintain exercise behaviour, behavioural strategies such as social support, goal setting and motivational interviewing should be implemented.

We suggest the following patient exclusion criteria/contraindications to exercise during haemodialysis:

- Less than three months after initiation of haemodialysis.
- Any uncontrolled medical condition including (but not limited to) infection or fever; recent (within 8 weeks) myocardial infarction or undiagnosed chest pain.
- Patient in class D (unstable condition) as per American Heart Association/American College of Sports Medicine Joint Position Statement: 1) unstable ischemia; 2) heart failure that is not compenstated;3) uncontrolled arrhythmias; 4) severe and symptomatic aortic stenosis; 5) hypertrophic cardiomyopathy or cardiomyopathy from recent myocarditis; 6) severe pulmonary hypertension; or 7) other conditions that could be aggravated by exercise (for example, resting systolic blood pressure >200 mm Hg or resting diastolic blood pressure >110 mm Hg; active or suspected myocarditis or pericarditis; suspected or known dissecting aneurysm; thrombophlebitis and recent systemic or pulmonary embolus).
- Symptomatic hyper- or hypotension.
- Signs and symptoms of deep vein thrombosis.
- Excessive inter-dialytic weight gain that severely impacts upon indices of fluid retention, e.g. blood pressure greater than 160/100; heart rate above 100 bpm; breathlessness at rest; or signs of substantial peripheral oedema.
- If diabetic, blood glucose above 16.7 mmol/L (300 mg/dL) AND patient is in ketosis (fruity breath, rapid breathing or shortness of breath, excessive thirst, frequent urination, stomach pain, nausea, vomiting, fatigue or confusion), is dehydrated, or is feeling unwell.
- In individuals taking insulin and/or insulin secretagogues, if hypoglycaemia below 5.5 mmol/L (100 mg/dL) is observed, and glucose containing dialysate fails to elevate blood glucose sufficiently, advise patient to eat or drink approximately 15g carbohydrate and re-assess blood glucose concentration after 20 min. Repeat until blood glucose exceeds 5.5 mmol/L.

We suggest the following safety monitoring:

- Prior to exercise, ask patient how they feel, record resting blood pressure and heart rate, and if diabetic, record blood glucose concentration.
- During exercise, monitor signs and ask patient to report symptoms of pain, excessive fatigue, altered consciousness, overheating, cyanosis, anxiety, severe breathlessness, chest pain, dizziness/light-headedness.
- If hypertensive, regularly check blood pressure during exercise. If values exceed 220/105 mm Hg, reduce exercise intensity or cease exercising until blood pressure reduces.
- Use a rating of perceived exertion scale (Borg CR100 or Borg RPE scales) and ensure exercise intensity does not provoke responses greater than 50/strong heavy on the Borg CR100 or 15/hard (heavy) on the Borg RPE scale

e. Post exercise, monitor blood pressure and heart rate until resting values are approximately obtained, observe patient for at least 20 min, and be aware of possibility of hypotension during remainder of the dialysis session.

## References

[1] Lowrie EG, Laird NM, Parker TF, Sargent JA (1981). Effect of the hemodialysis prescription of patient morbidity: report from the National Cooperative Dialysis Study. N Engl J Med.

[2] Gotch FA, Sargent JA (1985). A mechanistic analysis of the National Cooperative Dialysis Study (NCDS). Kidney Int.

[3] Held PJ, Port FK, Wolfe RA, Stannard DC, Carroll CE, Daugirdas JT (1996). The dose of hemodialysis and patient mortality. Kidney Int.

[4] Wolfe RA, Ashby VB, Daugirdas JT, Agodoa LY, Jones CA, Port FK (2000). Body size, dose of hemodialysis, and mortality. Am J Kidney Dis.

[5] Shinzato T, Nakai S, Akiba T, Yamazaki C, Sasaki R, Kitaoka T (1997). Survival in long-term haemodialysis patients: results from the annual survey of the Japanese Society for Dialysis Therapy. Nephrol Dial Transplant.

[6] Port FK, Ashby VB, Dhingra RK, Roys EC, Wolfe RA (2002). Dialysis dose and body mass index are strongly associated with survival in hemodialysis patients. J Am Soc Nephrol.

[7] Eknoyan G, Beck GJ, Cheung AK, Daugirdas JT, Greene T, Kusek JW (2002). Effect of dialysis dose and membrane flux in maintenance hemodialysis. N Engl J Med.

[8] Owen WF, Chertow GM, Lazarus JM, Lowrie EG (1998). Dose of hemodialysis and survival: differences by race and sex. JAMA..

[9] Port FK, Wolfe RA, Hulbert-Shearon TE, McCullough KP, Ashby VB, Held PJ (2004). High dialysis dose is associated with lower mortality among women but not among men. Am J Kidney Dis.

[10] Depner T, Daugirdas J, Greene T, Allon M, Beck G, Chumlea C (2004). Dialysis dose and the effect of gender and body size on outcome in the HEMO Study. Kidney Int.

[11] Daugirdas JT, Greene T, Chertow GM, Depner TA (2010). Can rescaling dose of dialysis to body surface area in the HEMO study explain the different responses to dose in women versus men? Clin J Am Soc Nephrol. Am Soc Nephrol.

[12] Cunningham JJ (1982). Body composition and resting metabolic rate: the myth of feminine metabolism. Am J Clin Nutr.

[13] Lowrie EG, Li Z, Ofsthun N, Lazarus JM (2005). The online measurement of hemodialysis dose (Kt): clinical outcome as a function of body surface area. Kidney Int.

[14] Lowrie EG, Li Z, Ofsthun N, Lazarus JM (2004). Measurement of dialyzer clearance, dialysis time, and body size: death risk relationships among patients. Kidney Int.

[15] Spalding EM, Chandna SM, Davenport A, Farrington K (2008). Kt/V underestimates the hemodialysis dose in women and small men. Kidney Int.

[16] Locatelli F, Buoncristiani U, Canaud B, Köhler H, Petitclerc T, Zucchelli P (2005). Dialysis dose and frequency. Nephrol Dial Transplant.

[17] Block GA, Klassen PS, Lazarus JM, Ofsthun N, Lowrie EG, Chertow GM (2004). Mineral metabolism, mortality, and morbidity in maintenance hemodialysis. J Am Soc Nephrol.

[18] Neirynck N, Vanholder R, Schepers E, Eloot S, Pletinck A, Glorieux G (2013). An update on uremic toxins. Int Urol Nephrol.

[19] Mactier RA, Madi AM, Allam BF (1997). Comparison of high-efficiency and standard haemodialysis providing equal urea clearances by partial and total dialysate quantification. Nephrol Dial Transplant.

[20] McGregor DO, Buttimore AL, Lynn KL, Nicholls MG, Jardine DL (2001). A Comparative Study of Blood Pressure Control with Short In-Center versus Long Home Hemodialysis. Blood Purif.

[21] Charra B, Chazot C, Jean G, Hurot J-M, Vanel T, Terrat J-C, et al. Long 3 x 8 hr dialysis: a three-decade summary. J Nephrol. 2003;16(Suppl 7):S64–9. 14733303

[22] Charra B, Calemard M, Laurent G (1996). Importance of treatment time and blood pressure control in achieving long-term survival on dialysis. Am J Nephrol.

[23] Held PJ, Levin NW, Bovbjerg RR, Pauly MV, Diamond LH (1991). Mortality and duration of hemodialysis treatment. JAMA..

[24] Marshall MR, Byrne BG, Kerr PG, McDonald SP (2006). Associations of hemodialysis dose and session length with mortality risk in Australian and New Zealand patients. Kidney Int.

[25] Tentori F, Zhang J, Li Y, Karaboyas A, Kerr P, Saran R (2012). Longer dialysis session length is associated with better intermediate outcomes and survival among patients on in-center three times per week hemodialysis: results from the Dialysis Outcomes and Practice Patterns Study (DOPPS). Nephrol Dial Transplant.

[26] Hassell DR, van der Sande FM, Kooman JP, Tordoir JP, Leunissen KM (2001). Optimizing dialysis dose by increasing blood flow rate in patients with reduced vascular-access flow rate. Am J Kidney Dis.

[27] Clark WR, Leypoldt JK, Henderson LW, Mueller BA, Scott MK, Vonesh EF (1999). Quantifying the effect of changes in the hemodialysis prescription on effective solute removal with a mathematical model. J Am Soc Nephrol.

[28] Ouseph R, Ward RA (2001). Increasing dialysate flow rate increases dialyzer urea mass transfer-area coefficients during clinical use. Am J Kidney Dis.

[29] Hauk M, Kuhlmann MK, Riegel W, Köhler H (2000). In vivo effects of dialysate flow rate on Kt/V in maintenance hemodialysis patients. Am J Kidney Dis.

[30] Leypoldt JK, Cheung AK (2001). Increases in mass transfer-area coefficients and urea Kt/V with increasing dialysate flow rate are greater for high-flux dialyzers. Am J Kidney Dis.

[31] Mandolfo S, Malberti F, Imbasciati E, Cogliati P, Gauly A (2003). Impact of blood and dialysate flow and surface on performance of new polysulfone hemodialysis dialyzers. Int J Artif Organs.

[32] Wei SS, Ellis PW, Magnusson MO, Paganini EP (1994). Effect of heparin modeling on delivered hemodialysis therapy. Am J Kidney Dis.

[33] Culleton BF (2007). Effect of frequent nocturnal hemodialysis vs conventional hemodialysis on left ventricular mass and quality of life: a randomized controlled trial. JAMA.

[34] Rocco MV (2011). The effects of frequent nocturnal home hemodialysis: the Frequent Hemodialysis Network Nocturnal Trial. Kidney Int.

[35] Chertow GM, Levin NW, Beck GJ, Depner TA, Eggers PW, FHN Trial Group (2010). In-center hemodialysis six times per week versus three times per week. N Engl J Med.

[36] Ok E (2011). Comparison of 4- and 8-h dialysis sessions in thrice-weekly in-centre haemodialysis: a prospective, case-controlled study. Nephrol Dial Transplant.

[37] Ipema KJ (2016). Nutritional Status in Nocturnal Hemodialysis Patients - A Systematic Review with Meta-Analysis. PLoS One.

[38] Wang W (2008). The Effect of Increasing Dialysis Dose in Overweight Hemodialysis Patients on Quality of Life: A 6-Week Randomized Crossover Trial. Am J Kidney Dis.

[39] Garg AX (2017). Patients receiving frequent hemodialysis have better health-related quality of life compared to patients receiving conventional hemodialysis. Kidney Int.

[40] Jardine MJ (2017). A Trial of Extending Hemodialysis Hours and Quality of Life. J Am Soc Nephrol.

[41] Rocco MV (2015). Long-term Effects of Frequent Nocturnal Hemodialysis on Mortality: The Frequent Hemodialysis Network (FHN) Nocturnal Trial. Am J Kidney Dis.

[42] Chertow GM, et al. Long-Term Effects of Frequent In-Center Hemodialysis. J Am Soc Nephrol. 2015;66(3):459–68. 10.1681/ASN.2015040426 PMC4884113 26467779

[43] Marshall MR (2016). Intensive Hemodialysis and Mortality Risk in Australian and New Zealand Populations. Am J Kidney Dis.

[44] Suri RS (2013). A multinational cohort study of in-center daily hemodialysis and patient survival. Kidney Int.

[45] Rivara MB (2016). Extended-hours hemodialysis is associated with lower mortality risk in patients with end-stage renal disease. Kidney Int.

[46] Daugirdas JT (2013). Effect of frequent hemodialysis on residual kidney function. Kidney Int.

[47] Suri RS (2014). Effects of frequent hemodialysis on perceived caregiver burden in the Frequent Hemodialysis Network trials. Clin J Am Soc Nephrol.

[48] NKF-DOQI clinical practice guidelines for hemodialysis adequacy (1997). National Kidney Foundation. Am J Kidney Dis.

[49] Hanson JA, Hulbert-Shearon TE, Ojo AO, Port FK, Wolfe RA, Agodoa LY (1999). Prescription of twice-weekly hemodialysis in the USA. Am J Nephrol.

[50] Panaput T, Thinkhamrop B, Domrongkitchaiporn S, Sirivongs D, Praderm L, Anukulanantachai J (2014). Dialysis Dose and Risk Factors for Death Among ESRD Patients Treated with Twice-Weekly Hemodialysis: A Prospective Cohort Study. Blood Purif.

[51] Park JI, Park JT, Kim Y-L, Kang S-W, Yang CW, Kim N-H (2016). Comparison of outcomes between the incremental and thrice-weekly initiation of hemodialysis: a propensity-matched study of a prospective cohort in Korea. Nephrol Dial Transplant.

[52] Lin Y-F, Huang J-W, Wu M-S, Chu T-S, Lin S-L, Chen Y-M (2009). Comparison of residual renal function in patients undergoing twice-weekly versus three-times-weekly haemodialysis. Nephrology (Carlton).

[53] Zhang M, Wang M, Li H, Yu P, Yuan L, Hao C (2014). Association of initial twice-weekly hemodialysis treatment with preservation of residual kidney function in ESRD patients. Am J Nephrol.

[54] Vilar E, Wellsted D, Chandna SM, Greenwood RN, Farrington K (2009). Residual renal function improves outcome in incremental haemodialysis despite reduced dialysis dose. Nephrol Dial Transplant.

[55] Lin X, Yan Y, Ni Z, Gu L, Zhu M, Dai H (2012). Clinical outcome of twice-weekly hemodialysis patients in shanghai. Blood Purif.

[56] Bieber B, Qian J, Anand S, Yan Y, Chen N, Wang M (2014). Two-times weekly hemodialysis in China: frequency, associated patient and treatment characteristics and Quality of Life in the China Dialysis Outcomes and Practice Patterns study. Nephrol Dial Transplant.

[57] Obi Y, Streja E, Rhee CM, Ravel V, Amin AN, Cupisti A (2016). Incremental Hemodialysis, Residual Kidney Function, and Mortality Risk in Incident Dialysis Patients: A Cohort Study. Am J Kidney Dis.

[58] Lucas MF, Teruel JL, Ruíz-Roso G, Díaz M, Raoch V, Caravaca F, et al. Incremental Hemodialysis Schedule in Patients with Higher Residual Renal Function at the Start of Dialysis. Adv Nephrol. 2014;37:1–6.

[59] Fernández-Lucas M, Teruel-Briones JL, Gomis-Couto A, Villacorta-Pérez J, Quereda-Rodríguez-Navarro C (2012). Maintaining residual renal function in patients on haemodialysis: 5-year experience using a progressively increasing dialysis regimen. Nefrologia..

[60] Vandecasteele SJ, Kurella TM (2014). A patient-centered vision of care for ESRD: dialysis as a bridging treatment or as a final destination?. J Am Soc Nephrol.

[61] Tattersall J, Farrington K, Gentile G (2018). Is Kt/V useful in elderly dialysis patients? Pro and Con arguments. Nephrol Dial Transplant.

[62] Kurella Tamura M, Covinsky KE, Chertow GM, Yaffe K, Landefeld CS, McCulloch CE (2009). Functional status of elderly adults before and after initiation of dialysis. N Engl J Med.

[63] Sharma A (2001). Reassessing haemodialysis adequacy in children: the case for more. Pediatr Nephrol.

[64] Goldstein SL (2004). Adequacy of dialysis in children: does small solute clearance really matter?. Pediatr Nephrol.

[65] Bell L, Espinosa P (2003). Intensive in center hemodialysis for children: a case for longer dialysis duration. Hemodial Int.

[66] Fischbach M, Terzic J, Laugel V, Dheu C, Menouer S, Helms P, Livolsi A (2004). Daily on line hemodiafiltration: a pilot experience in children. Nephrol Dial Transplant.

[67] Coulthard MG, Sharp J (2001). Hemodialysis in infants: theoretical limitations, and singles versus double lumen lines. Pediatr Nephrol.

[68] Saliem S, Patenaude V, Abenhaim HA (2016). Pregnancy outcomes among renal transplant recipients and patients with end-stage renal disease on dialysis. J Perinat Med.

[69] Barua M, Hladunewich M, Keunen J, Pierratos A, McFarlane P, Sood M, Chan CT (2008). Successful pregnancies on nocturnal home hemodialysis. Clin J Am Soc Nephrol.

[70] Thompson S, Marnoch CA, Habib S, Robinson H, Pauly RP (2011). A successful term pregnancy using in-center intensive quotidian hemodialysis. Hemodial Int.

[71] Piccoli GB, Minelli F, Versino E, Cabiddu G, Attini R, Vigotti FN, Rolfo A, Giuffrida D, Colombi N, Pani A, Todros T (2016). Pregnancy in dialysis patients in the new millennium: a systematic review and meta-regression analysis correlating dialysis schedules and pregnancy outcomes. Nephrol Dial Transplant.

[72] Hladunewich MA, Hou S, Odutayo A, Cornelis T, Pierratos A, Goldstein M, Tennankore K, Keunen J, Hui D, Chan CT (2014). Intensive hemodialysis associates with improved pregnancy outcomes: a Canadian and United States cohort comparison. J Am Soc Nephrol.

[73] Daugirdas JT, Greene T, Rocco MV, Kaysen GA, Depner TA, Levin NW, Chertow GM, Ornt DB, Raimann JG, Larive B (2013). Kliger AS; FHN Trial Group. Effect of frequent hemodialysis on residual kidney function. Kidney Int.

[74] Hladunewich M, Schatell D (2016). Intensive dialysis and pregnancy. Hemodial Int.

[75] Clark WR, Gao D, Neri M, Ronco C (2017). Solute Transport in Hemodialysis: Advances and Limitations of Current Membrane Technology. Contrib Nephrol.

[76] Sternby JP, Nilsson A, Garred LJ (2005). Diffusive-Convective Mass Transfer Rates for Solutes Present on Both Sides of a Dialyzer Membrane. ASAIO J.

[77] Locatelli F, Altieri P, Andrulli S, Sau G, Bolasco P, Pedrini LA, Basile C, David S, Gazzanelli L, Tampieri G, Isola E, Marzolla O, Memoli B, Ganadu M, Reina E, Bertoli S, Ferrara R, Casu D, Logias F, Tarchini R, Mattana G, Passaghe M, Fundoni G, Villa G, Di Iorio BR, Pontoriero G, Zoccali C (2014). Phosphate levels in patients treated with low-flux haemodialysis, pre-dilution haemofiltration and haemodiafiltration: post hoc analysis of a multicentre, randomized and controlled trial. Nephrol Dial Transplant.

[78] Roumelioti ME, Trietley G, Nolin TD, Ng YH, Xu Z, Alaini A, Figueroa R, Unruh ML, Argyropoulos CP (2017). Beta-2 microglobulin clearance in high-flux dialysis and convective dialysis modalities: a meta-analysis of published studies. Nephrol Dial Transplant.

[79] Cornelis T, Eloot S, Vanholder R, Glorieux G, van der Sande FM, Scheijen JL, Leunissen KM, Kooman JP, Schalkwijk CG (2015). Protein-bound uraemic toxins, dicarbonyl stress and advanced glycation end products in conventional and extended haemodialysis and haemodiafiltration. Nephrol Dial Transplant.

[80] Cheung AK, Levin NW, Greene T, Agodoa L, Bailey J, Beck G, Clark W, Levey AS, Leypoldt JK, Ornt DB, Rocco MV, Schulman G, Schwab S, Teehan B, Eknoyan G (2003). Effects of high-flux hemodialysis on clinical outcomes: results of the HEMO study. J Am Soc Nephrol.

[81] Locatelli F, Martin-Malo A, Hannedouche T, Loureiro A, Papadimitriou M, Wizemann V, Jacobson SH, Czekalski S, Ronco C (2009). Vanholder R; Membrane Permeability Outcome (MPO) Study Group. Effect of membrane permeability on survival of hemodialysis patients. J Am Soc Nephrol.

[82] Palmer SC, Rabindranath KS, Craig JC, Roderick PJ, Locatelli F, Strippoli GF. High-flux versus low-flux membranes for end-stage kidney disease. Cochrane Database Syst Rev. 2012;(9):CD005016. 10.1002/14651858.CD005016.pub2 PMC6956628 22972082

[83] Schepers E, Glorieux G, Eloot S, Hulko M, Boschetti-de-Fierro A, Beck W, Krause B, Van Biesen W (2018). Assessment of the association between increasing membrane pore size and endotoxin permeability using a novel experimental dialysis simulation set-up. BMC Nephrol.

[84] Kim HW, Kim SH, Kim YO, Jin DC, Song HC, Choi EJ, Kim YL, Kim YS, Kang SW, Kim NH, Yang CW, Kim YK. Comparison of the impact of high-flux dialysis on mortality in hemodialysis patients with and without residual renal function. PLoS One. 2014;9(6):e97184. Published 2014 June 6. 10.1371/journal.pone.0097184. 10.1371/journal.pone.0097184 PMC4048156 24906205

[85] Grooteman MP, van den Dorpel MA, Bots ML (2012). Effect of online hemodiafiltration on all-cause mortality and cardiovascular outcomes. J Am Soc Nephrol.

[86] Ok E, Asci G, Toz H (2013). Mortality and cardiovascular events in online haemodiafiltration (OL-HDF) compared with high-flux dialysis: results from the Turkish OL-HDF Study. Nephrol Dial Transplant.

[87] Morena M, Jaussent A, Chalabi L, Leray-Moragues H, Chenine L, Debure A, Thibaudin D, Azzouz L, Patrier L, Maurice F, Nicoud P, Durand C, Seigneuric B, Dupuy AM, Picot MC, Cristol JP (2017). Canaud B; FRENCHIE Study Investigators. Treatment tolerance and patient-reported outcomes favor online hemodiafiltration compared to high-flux hemodialysis in the elderly. Kidney Int.

[88] Maduell F, Moreso F, Pons M (2013). High-efficiency postdilution online hemodiafiltration reduces all-cause mortality in hemodialysis patients. J Am Soc Nephrol.

[89] Nubé MJ, Peters SAE, Blankestijn PJ, Canaud B, Davenport A, Grooteman MPC, Asci G, Locatelli F, Maduell F, Morena M, Ok E, Torres F (2017). Bots ML; HDF Pooling Project investigators. Mortality reduction by post-dilution online-haemodiafiltration: a cause-specific analysis. Nephrol Dial Transplant.

[90] Nistor I, Palmer SC, Craig JC, Saglimbene V, Vecchio M, Covic A, Strippoli GF. Haemodiafiltration, haemofiltration and haemodialysis for end-stage kidney disease. Cochrane Database Syst Rev. 2015;(5):CD006258. 10.1002/14651858.CD006258.pub2 PMC10766139 25993563

[91] Locatelli F, Karaboyas A, Pisoni RL, Robinson BM, Fort J, Vanholder R, Rayner HC, Kleophas W, Jacobson SH, Combe C, Port FK, Tentori F. Mortality risk in patients on hemodiafiltration versus hemodialysis: a ‘real-world’ comparison from the DOPPS. Nephrol Dial Transplant. 2017;33(4):683–9. 10.1093/ndt/gfx277 PMC5888924 29040687

[92] Locatelli F, Altieri P, Andrulli S, Bolasco P, Sau G, Pedrini LA, Basile C, David S, Feriani M, Montagna DiIorio BR, Memoli B, Cravero R, Battaglia G, Zoccali C (2010). Hemofiltration and Hemodiafiltration Reduce Intradialytic Hypotension in ESRD. J Am Soc Nephrol.

[93] Flythe JE (2015). Associations of Posthemodialysis Weights above and below Target Weight with All-Cause and Cardiovascular Mortality. CJASN May.

[94] Sands JJ, Usvyat LA, Sullivan T (2014). Intradialytic hypotension: frequency, sources of variation and correlation with clinical outcome. Hemodial Int.

[95] Leung KCW (2017). Randomized Crossover Trial of Blood Volume Monitoring-Guided Ultrafiltration Biofeedback to Reduce Intradialytic Hypotensive Episodes with Hemodialysis. Clin J Am Soc Nephrol.

[96] Nur E (2013). Effect of Fluid Management Guided by Bioimpedance Spectroscopy on Cardiovascular Parameters in Hemodialysis Patients: A Randomized Controlled Trial. AJKD.

[97] Saran R (2006). Longer treatment time and slower ultrafiltration in hemodialysis: associations with reduced mortality in the DOPPS. Kidney Int.

[98] Flythe JE (2011). Rapid fluid removal during dialysis is associated with cardiovascular morbidity and mortality. Kidney Int.

[99] Gümrükçüoğlu HA, Arı E, Akyol A, Akdağ S, Simşek H, Sahin M, Güneş Y, Tuncer M (2012). Effects of lowering dialysate sodium on carotid artery atherosclerosis and endothelial dysfunction in maintenance hemodialysis patients. Int Urol Nephrol.

[100] Hecking M (2012). Dialysate sodium concentration and the association with interdialytic weight gain, hospitalization, and mortality. Clin J Am Soc Nephrol.

[101] van der Sande FM, Rosales LM, Brener Z (2005). Effect of ultrafiltration on thermal variables, skin temperature, skin blood flow, and energy expenditure during ultrapure hemodialysis. J Am Soc Nephrol.

[102] Selby NM, McIntyre CW (2006). A systematic review of the clinical effects of reducing dialysate fluid temperature. Nephrol Dial Transplant.

[103] Mustafa RA, Bdair F, Akl EA (2016). Effect of Lowering the Dialysate Temperature in Chronic Hemodialysis: A Systematic Review and Meta-Analysis. Clin J Am Soc Nephrol.

[104] Maggiore Q (2002). Study Group of Thermal Balance and Vascular Stability: The effects of control of thermal balance on vascular stability in hemodialysis patients: Results of the European randomized clinical trial. Am J Kidney Dis.

[105] Fine A, Penner B (1996). The protective effect of cool dialysate is dependent on patients’ predialysis temperature. Am J Kidney Dis.

[106] Santos SF, Peixoto AJ, Perazella MA (2012). How should we manage adverse intradialytic blood pressure changes?. Adv Chronic Kidney Dis.

[107] Knoll GA, Grabowski JA, Dervin GF, O'Rourke K (2004). A randomized, controlled trial of albumin versus saline for the treatment of intradialytic hypotension. J Am Soc Nephrol.

[108] Prakash S, Garg AX, Heidenheim AP, House AA (2004). Midodrine appears to be safe and effective for dialysis-induced hypotension: a systematic review. Nephrol Dial Transplant.

[109] Fischbach M, Zita N, Birmele B, Geisert J (1998). Sequential hypertonic dialysis (SHD) in children. Pediatr Nephrol.

[110] Fischbach M, Mengus L, Simeoni U, Durringer R, Mark J, De Geeter B, Hamel G, Geisert J (1991). Dialyse à double profil: ultrafiltration et sodium variables. Description et validation clinique chez l’enfant. Néphrologie.

[111] Huang CW, Lee MJ, Lee PT, Hsu CY, Huang WC, Chen CL, Chou KJ, Fang HC (2015). Low Potassium Dialysate as a Protective Factor of Sudden Cardiac Death in Hemodialysis Patients with Hyperkalemia. PLoS One.

[112] Hwang JC, Wang CT, Chen CA, Chen HC (2011). Hypokalemia is associated with increased mortality rate in chronic hemodialysis patients. Blood Purif.

[113] Kovesdy CP, Regidor DL, Mehrotra R, Jing J, McAllister CJ, Greenland S, Kopple JD, Kalantar-Zadeh K (2007). Serum and dialysate potassium concentrations and survival in hemodialysis patients. Clin J Am Soc Nephrol.

[114] Pun PH, Lehrich RW, Honeycutt EF, Herzog CA, Middleton JP (2011). Modifiable risk factors associated with sudden cardiac arrest within hemodialysis clinics. Kidney Int.

[115] Karnik JA, Young BS, Lew NL, Herget M, Dubinsky C, Lazarus JM, Chertow GM (2001). Cardiac arrest and sudden death in dialysis units. Kidney Int.

[116] Jadoul M, Thumma J, Fuller DS, Tentori F, Li Y, Morgenstern H, Mendelssohn D, Tomo T, Ethier J, Port F, Robinson BM (2012). Modifiable practices associated with sudden death among hemodialysis patients in the Dialysis Outcomes and Practice Patterns Study. Clin J Am Soc Nephrol.

[117] Karaboyas A, Zee J, Brunelli SM, Usvyat LA, Weiner DE, Maddux FW, Nissenson AR, Jadoul M, Locatelli F, Winkelmayer WC, Port FK, Robinson BM, Tentori F (2017). Dialysate Potassium, Serum Potassium, Mortality, and Arrhythmia Events in Hemodialysis: Results From the Dialysis Outcomes and Practice Patterns Study (DOPPS). Am J Kidney Dis.

[118] Gutzwiller JP, Schneditz D, Huber AR, Schindler C, Garbani E, Zehnder CE (2003). Increasing blood flow increases Kt/V(urea) and potassium removal but fails to improve phosphate removal. Clin Nephrol.

[119] Tucker B, Moledina DG (2016). We Use Dialysate Potassium Levels That Are Too Low in Hemodialysis. Semin Dial.

[120] Noureddine L, Dixon BS (2015). Complications and management of hyperkalemia: implications for the use of the novel cation exchangers zirconium cyclosilicate and patiromer. Clin Invest (Lond).

[121] Agar BU, Culleton BF, Fluck R, Leypoldt JK (2015). Potassium kinetics during hemodialysis. Hemodial Int.

[122] Di Iorio B, Torraca S, Piscopo C, Sirico ML, Di Micco L, Pota A, Tartaglia D, Berardino L, Morrone LF, Russo D (2012). Dialysate bath and QTc interval in patients on chronic maintenance hemodialysis: pilot study of single dialysis effects. J Nephrol.

[123] Tentori F, Karaboyas A, Robinson BM, Morgenstern H, Zhang J, Sen A, Ikizler TA, Rayner H, Fissell RB, Vanholder R, Tomo T, Port FK (2013). Association of dialysate bicarbonate concentration with mortality in the Dialysis Outcomes and Practice Patterns Study (DOPPS). Am J Kidney Dis.

[124] https://ajkdblog.org/2013/07/25/dialysis-bicarbonate-concentration-do-we-know-the-right-amount/. Accessed June 2018.

[125] Lew SQ, Kohn OF, Cheng YL, Kjellstrand CM, Ing TS (2017). Three-Stream, Bicarbonate-Based Hemodialysis Solution Delivery System Revisited: With an Emphasis on Some Aspects of Acid-Base Principles. Artif Organs.

[126] Kraut JA, Madias NE (2016). Metabolic Acidosis of CKD: An Update. Am J Kidney Dis.

[127] Lowrie EG, Lew NL (1990). Death risk in hemodialysis patients: The predictive value of commonly measured variables and an evaluation of death rate differences between facilities. Am J Kidney Dis.

[128] Bommer J, Locatelli F, Satayathum S, Keen ML, Goodkin DA, Saito A, Akiba T, Port FK, Young EW (2004). Association of predialysis serum bicarbonate levels with risk of mortality and hospitalization in the Dialysis Outcomes and Practice Patterns Study (DOPPS). Am J Kidney Dis.

[129] Wu DY, Shinaberger CS, Regidor DL, McAllister CJ, Kopple JD, Kalantar-Zadeh K (2006). Association between serum bicarbonate and death in hemodialysis patients: is it better to be acidotic or alkalotic? Clin J Am Soc Nephrol. Jan.

[130] Fouque D, Vennegoor M, Ter Wee P, Wanner C, Basci A, Canaud B, Vanholder R. EBPG Guideline on Nutrition. Nephrol Dial Transplant. 2007;22(Suppl 2):ii45–ii87. 10.1093/ndt/gfm020 17507426

[131] KDOQI Nutrition in Chronic Renal Failure. Am J Kidney Dis. 2000;35(6 Suppl 2):S1–140. 10.1053/ajkd.2000.v35.aajkd03517 10895784

[132] http://www.renalweb.com/writings/alkalosis/WithinFMC.htm. Accessed June 2018.

[133] Heguilén RM, Sciurano C, Bellusci AD, Fried P, Mittelman G, Rosa Diez G, Bernasconi AR (2005). The faster potassium-lowering effect of high dialysate bicarbonate concentrations in chronic haemodialysis patients. Nephrol Dial Transplant.

[134] http://www.mayomedicallaboratories.com/test-catalog/Clinical+and+Interpretive/876. Accessed June 2018.

[135] Bandi ZL (1981). Estimation, prevention, and quality control of carbon dioxide loss during aerobic sample processing. Clin Chem.

[136] Brunelli SM, Goldfarb S (2007). Hypophosphatemia: clinical consequences and management. J Am Soc Nephrol.

[137] Lertdumrongluk P, Rhee CM, Park J, Lau WL, Moradi H, Jing J, Molnar MZ, Brunelli SM, Nissenson AR, Kovesdy CP, Kalantar-Zadeh K (2013). Association of serum phosphorus concentration with mortality in elderly and nonelderly hemodialysis patients. J Ren Nutr.

[138] Hanudel MR, Froch L, Gales B, Jüppner H, Salusky IB. Fractures and Osteomalacia in a Patient Treated With Frequent Home Hemodialysis. Am J Kidney Dis. 2017; [Epub ahead of print]. 10.1053/j.ajkd.2017.03.015 PMC5572082 28495360

[139] Ing TS, Chebrolu SB, Cheng YL, Yu AW, Choi P, Kjellstrand CM (2003). Phosphorus-enriched hemodialysates: formulations and clinical use. Hemodial Int.

[140] Sam R, Tang HL, Kjellstrand CM, Ing TS (2015). Preventing/treating hypophosphatemia by adding phosphate to the dialysate. Int J Artif Organs.

[141] Godaly G, Carlsson O, Broman M (2016). Phoxilium(®) reduces hypophosphataemia and magnesium supplementation during continuous renal replacement therapy. Clin Kidney J.

[142] Cleen Ready-to-Use 21.4g/9.4g Enema PIL. 2013. www.medicines.org.uk/emc/PIL.20176.latest.pdf. Accessed Aug 2017.

[143] Pierratos A (1999). Nocturnal home haemodialysis: an update on a 5-year experience. Nephrol Dial Transplant.

[144] Su WS, Lekas P, Carlisle EJ, Cowin R, Bellamy J, Margetts PJ, Brimble KS, Clase CM, Gangji AS (2011). Management of hypophosphatemia in nocturnal hemodialysis with phosphate-containing enema: a technical study. Hemodial Int.

[145] Ebah LM, Akhtar M, Wilde I, Hookway G, Vincent M, Reeves C, Denton J, Woods J, Mitra S (2012). Phosphate enrichment of dialysate for use in standard and extended haemodialysis. Blood Purif.

[146] Oh J, Wunsch R, Turzer M, Bahner M, Raggi P, Querfeld U, Mehls O, Schaefer F (2002). Advanced coronary and carotid arteriopathy in young adults with childhood onset chronic renal failure. Circulation.

[147] Goodman WG, Goldin J, Kuizon BD, Yoon C, Gales B, Sider D, Wang Y, Chung J, Emerick A, Greaser L, Elashoff RM, Salusky IB (2000). Coronary-artery calcification in young adults with end-stage renal disease who are undergoing dialysis. New Engl J Med.

[148] Fischbach M, Terzic J, Menouer S, Provot E, Bergere V (2001). Hemodialysis in children: principles and practice. Semin Nephrol.

[149] Ouseph R, Ward RA (2000). Anticoagulation for intermittent haemodialysis. Semin Dial.

[150] Brunelli SM, Cohen DE, Marlowe G, Liu D, Njord L, Van Wyck D, Aronoff G. Safety and efficacy of heparin during dialysis in the context of systemic anticoagulant and antiplatelet medications. J Nephrol. 2019. 10.1007/s40620-018-00576-w PMID: 30604148. 10.1007/s40620-018-00576-w 30604148

[151] Swartz RD (1981). Hemorrhage during high-risk hemodialysis using controlled heparinization. Nephron..

[152] European Best Renal Practice Guidelines for haemodialysis Part 1. Nephrol Dial Transplant. 2002;17:Supplement 7 S1-S111

[153] Dhondt A, Pauwels R, Devreese K, Eloot S, Glorieux G, Vanholder R (2015). Where and When To Inject Low Molecular Weight Heparin in Hemodiafiltration? A Cross Over Randomised Trial. PLoS One.

[154] Leu JG, Chiang SS, Lin SM, Pai JK, Jiang WW (2000). Low molecular weight heparin in haemodialysis patients with a bleeding tendency. Nephron..

[155] Lim W, Cook DJ, Crowther MA (2004). Safety and efficacy of low molecular weight heparins for haemodialysis in patients with end-stage renal failure: a meta-analysis of randomised trials. J Am Soc Nephrol.

[156] Palamaner Subash Shantha G, Kumar AA, Sethi M, Khanna RC, Pancholy SB (2015). Efficacy and safety of low molecular weight heparin compared to unfractionated heparin for chronic outpatient hemodialysis in end stage renal disease: systematic review and meta-analysis. PeerJ.

[157] Shen JI, Winkelmayer WC (2012). Use and safety of unfractionated heparin for anticoagulation during maintenance haemodialysis. Am J Kidney Dis.

[158] Lazrak HH, René É, Elftouh N, Leblanc M, Lafrance JP (2017). Safety of low-molecular-weight heparin compared to unfractionated heparin in hemodialysis: a systematic review and meta-analysis. BMC Nephrol.

[159] Sahota S, Rodby R (2014). Inpatient haemodialysis without anticoagulation in adults. Clin Kidney J.

[160] Guéry B, Alberti C, Servais A, Harrami E, Bererhi L, Zins B, Touam M, Joly D (2014). Hemodialysis without systemic anticoagulation: a prospective randomized trial to evaluate 3 strategies in patients at risk of bleeding. PLoS One.

[161] Lavaud S, Paris B, Maheut H, Randoux C, Renaux JL, Rieu P, Chanard J (2005). Assessment of the heparin-binding AN69 ST hemodialysis membrane: II. Clinical studies without heparin administration. ASAIO J.

[162] Laville M, Dorval M, Fort Ros J, Fay R, Cridlig J, Nortier JL, Juillard L, Dębska-Ślizień A, Fernández Lorente L, Thibaudin D, Franssen C, Schulz M, Moureau F, Loughraieb N, Rossignol P (2014). Results of the HepZero study comparing heparin-grafted membrane and standard care show that heparin-grafted dialyzer is safe and easy to use for heparin-free dialysis. Kidney Int.

[163] Kossmann RJ, Gonzales A, Callan R, Ahmad S (2009). Increased efficiency of hemodialysis with citrate dialysate: a prospective controlled study. Clin J Am Soc Nephrol.

[164] Hanevold C, Lu S, Yonekawa K (2010). Utility of citrate dialysate in management of acute kidney injury in children. Hemodial Int.

[165] Sands JJ, Kotanko P, Segal JH, Ho CH, Usvat L, Young A, Carter M, Sergeyeva O, Korth L, Maunsell E, Zhu Y, Krishnan M, Diaz-Buxo JA (2012). Effects of citrate acid concentrate (citrasate®) on heparin N requirements and hemodialysis adequacy: a multicenter, prospective noninferiority trial. Blood Purif.

[166] Kreuzer M, Bonzel KE, Büscher R, Offner G, Ehrich JH, Pape L (2010). Regional citrate anticoagulation is safe in intermittent high-flux haemodialysis treatment of children and adolescents with an increased risk of bleeding. Nephrol Dial Transplant.

[167] Swartz RD, Flamenbaum W, Dubrow A, Hall JC, Crow JW, Cato A (1988). Epoprostenol (PGI2, prostacyclin) during high-risk hemodialysis: preventing further bleeding complications. J Clin Pharmacol.

[168] Wright S, Steinwandel U, Ferrari P (2011). Citrate anticoagulation using ACD solution A during long-term haemodialysis. Nephrology (Carlton).

[169] Warkentin TE (2015). Heparin-induced thrombocytopenia in critically ill patients. Semin Thromb Hemost.

[170] Nand S, Wong W, Yuen B, Yetter A, Schmulbach E, Gross FS (1997). Heparin-induced thrombocytopenia with thrombosis: incidence, analysis of risk factors, and clinical outcomes in 108 consecutive patients treated at a single institution. Am J Hematol.

[171] Linkins LA, Bates SM, Lee AY, Heddle NM, Wang G, Warkentin TE (2015). Combination of 4Ts score and PF4/H-PaGIA for diagnosis and management of heparin-induced thrombocytopenia: prospective cohort study. Blood.

[172] Scully M, Gates C, Neave L. How we manage patients with heparin induced thrombocytopenia. Br J Haematol. 2016. 10.1111/bjh.14102. 10.1111/bjh.14102 27097741

[173] Murray PT, Hursting MJ (2006). Heparin-induced thrombocytopenia in patients administered heparin solely for hemodialysis. Ren Fail.

[174] Magnani HN (2010). A review of 122 published outcomes of danaparoid anticoagulation for intermittent haemodialysis. Thromb Res.

[175] Mahieu E, Claes K, Jacquemin M, Evenepoel P, Op De Beek K, Bogaert AM, Kuypers D, Verhamme P, Meijers B (2013). Anticoagulation with fondaparinux for hemodiafiltration in patients with heparin-induced thrombocytopenia: dose-finding study and safety evaluation. Artif Organs.

[176] Dhakal P, Giri S, Pathak R, Bhatt VR (2015). Heparin Reexposure in Patients With a History of Heparin-Induced Thrombocytopenia. Clin Appl Thromb Hemost.

[177] Awobusuyi JO, Mapayi FA, Adedolapo A (2008). Blood loss during vascular access cannulation: Quantification using the weighed gauze and drape method. Haemodial Int.

[178] Lin CL, Chen HY, Huang SC, Hsu SP, Pai MF, Peng YS, Chiu YL (2014). Increased blood loss from access cannulation site during hemodialysis is associated with anemia and arteriovenous graft use. Ther Apher Dial.

[179] Tsai WC, Chen HY, Lin CL, Huang SC, Hsu SP, Pai MF, Peng YS, Chiu YL (2015). Excessive cannulation site bleeding predicts long term all-cause mortality in chronic hemodialysis patients. Ther Apher Dial.

[180] Kalantar-Zadeh K, Streia E, Miller JE, Nissenson AR (2009). Intravenous iron versus erythropoietin stimulating agents: Friends or foes in treating chronic kidney disease anemia. Adv Chronic Kid Dis Mar.

[181] Verhallen AM, Kooistra MP, van Jaarsveld BC (2007). Cannulating in haemodialysis: rope-ladder or buttonhole technique?. Nephrol Dial Transplant.

[182] McCann M, Einarsdóttir H, Van Waeleghem JP, Murphy F, Sedgewick J (2008). J Ren Care.

[183] Fruits RG, Kinney R, Hegeman T (1992). Maintaining iron balance by decreasing blood loss. ANNA J.

[184] Kalocheretis P, Vlamis I, Belesi C, Makriniotou I, Zerbala S, Savidou E, Zorbas S, Arvanitis N, Iatrou C (2006). Residual blood loss in single use dialyzers: effect of different membranes and flux. Int J Artif Organs.

[185] Daugirdas JT, Tattersall JE (2010). Automated monitoring of hemodialysis adequacy by dialysis machines: potential benefits to patients and cost savings. Kidney Int.

[186] Van Waeleghem JP, Chamney M, Lindley EJ, Pancírová J (2008). Venous needle dislodgement: how to minimise the risks. J Ren Care.

[187] Axley B, Speranza-Reid J, Williams H (2012). Venous needle dislodgement in patients on hemodialysis. Nephrol Nurs J.

[188] Zeigler SA (2007). Prevent dangerous hemodialysis catheter disconnections. Nursing..

[189] International Standard IEC 60601-2-16 (2008). Medical electrical equipment- part 2-16: Particular requirements for the safety of haemodialysis, haemodiafiltration and haemofiltration equipment.

[190] Allcock K, Jagannathan B, Hood CJ, Marshall MR (2012). Exsanguination of a home hemodialysis patient as a result of misconnected blood-lines during the wash back procedure: a case report. BMC Nephrol.

[191] Ribitsch W, Schilcher G, Hafner-Giessauf H, Krisper P, Horina JH, Rosenkranz AR, Schneditz D (2014). Prevalence of detectable venous pressure drops expected with venous needle dislodgement. Semin Dial.

[192] Polaschegg HD (2010). Venous needle dislodgement: the pitfalls of venous pressure measurement and possible alternatives, a review. J Ren Care.

[193] Garrick R, Morey R (2015). Dialysis Facility Safety: Processes and Opportunities. Semin Dial.

[194] Takeuchi A, Miwa T, Shirataka M, Sawada M, Imaizumi H, Sugibuchi H, Ikeda N (2010). Non-cladding optical fiber is available for detecting blood or liquids. J Clin Monit Comput.

[195] NHS PASA Centre for Evidence-based Purchasing. Evidence Review: Redsense blood loss detection device for venous needle dislodgement monitoring in haemodialysis. 2009. Available at https://renal.org/wp-content/uploads/2017/07/redsense-cep08050-mar-09.pdf.

[196] Pauly RP, Eastwood DO, Marshall MR (2015). Patient safety in home hemodialysis: quality assurance and serious adverse events in the home setting. Hemodial Int.

[197] Wong B, Zimmerman D, Reintjes F, Courtney M, Klarenbach S, Dowling G, Pauly RP (2014). Procedure-related serious adverse events among home hemodialysis patients: a quality assurance perspective. Am J Kidney Dis.

[198] Hawley CM, Jeffries J, Nearhos J, Van Eps C (2008). Complications of home hemodialysis. Hemodial Int.

[199] Tennankore KK, d'Gama C, Faratro R, Fung S, Wong E, Chan CT (2015). Adverse technical events in home hemodialysis. Am J Kidney Dis.

[200] Ahlmén J, Gydell KH, Hadimeri H, Hernandez I, Rogland B, Strömbom U (2008). A new safety device for hemodialysis. Hemodial Int.

[201] Nicholls AJ, Platts MM (1982). Anaphylactoid reactions due to haemodialysis, haemofiltration, or membrane plasma separation. Br Med J (Clin Res Ed).

[202] Nicholls AJ (1987). Hypersensitivity to hemodialysis: the United Kingdom experience. Artif Organs.

[203] Grammer LC, Roberts M, Nicholls AJ, Platts MM, Patterson R (1984). IgE against ethylene oxide-altered human serum albumin in patients who have had acute dialysis reactions. J Allergy Clin Immunol.

[204] Schaefer RM, Fink E, Schaefer L, Barkhausen R, Kulzer P, Heidland A (1993). Role of bradykinin in anaphylactoid reactions during hemodialysis with AN69 dialyzers. Am J Nephrol.

[205] Amore A, Guarnieri G, Atti M, Schena FP, Coppo R (1999). Use of alkaline rinsing solution to prevent hypersensitivity reactions during hemodialysis: data from a multicentre retrospective analysis. J Nephrol.

[206] Sánchez-Villanueva RJ, González E, Quirce S, Díaz R, Alvarez L, Menéndez D, Rodríguez-Gayo L, Bajo MA, Selgas R (2014). Hypersensitivity reactions to synthetic haemodialysis membranes. Nefrologia..

[207] Hildebrand S, Corbett R, Duncan N, Ashby D (2016). Increased prevalence of eosinophilia in a hemodialysis population: Longitudinal and case control studies. Hemodial Int.

[208] Kiaii M, Djurdjev O, Farah M, Levin A, Jung B, MacRae J (2011). Use of electron-beam sterilized hemodialysis membranes and risk of thrombocytopenia. JAMA..

[209] Finkelstein FO, Schiller B, Daoui R, Gehr TW, Kraus MA, Lea J (2012). At-home short daily hemodialysis improves the long-term health-related quality of life. Kidney Int.

[210] Jefferies HJ, Virk B, Schiller B, Moran J, McIntyre CW (2011). Frequent hemodialysis schedules are associated with reduced levels of dialysis-induced cardiac injury (myocardial stunning). Clin J Am Soc Nephrol CJASN.

[211] Tennankore KK, Chan CT, Curran SP (2012). Intensive home haemodialysis: benefits and barriers. Nat Rev Nephrol.

[212] Beard C. No place like home: Increasing access to home dialysis. Available from: http://www.nwcscnsenate.nhs.uk/files/2814/2919/9869/16-04-2015_1557_680.pdf. Accessed June 2018.

[213] Tabinor M, Casula A, Wilkie M, Davies S, Caskey F, Lambie M (2017). UK Renal Registry 19th Annual Report: Chapter 13 Home Therapies in 2015: National and Centre-specific Analyses. Nephron..

[214] Mitra S. NICE Guidance: Improving Choice for Kidney Patients and Carers - Increasing access to home dialysis [Internet]. NICE. 2011; Available from: https://www.nice.org.uk/Contents/Item/Display/30787. [cited 2018 Apr 29].

[215] Farrington K, Warwick G. Renal Association Clinical Practice Guideline on Planning, Initiating and Withdrawal of RenalReplacement Therapy. Nephron Clin Pract. 2011;118(Suppl 1):c189–208. 10.1159/000328069. 10.1159/000328069 21555896

[216] Jenkins K, Adams A (2015). National home adaptation and reimbursement guidance. J Ren Nurs.

[217] Improvement Hub » Improving Choice for Kidney Patients: Five STEPS Toolkit to Home Haemodialysis [Internet]. Available from: https://www.england.nhs.uk/improvement-hub/publication/improving-choice-for-kidney-patients-five-steps-toolkit-to-home-haemodialysis/. [cited 2018 Apr 29].

[218] The Renal Team A Multi-Professional Renal Workforce Plan For Adults and Children with Renal Disease.pdf [Internet]. Available from: http://www.wales.nhs.uk/sites3/documents/434/Workforce.pdf. [cited 2018 Apr 29].

[219] Implementing Hemodialysis in the Home: A practical manual [Internet]. Available from: http://www.ishd.org/library/pdfs/HomeHemo_AllModules2.pdf. [cited 2018 Apr 29].

[220] Clinical Practice Guideline Peritoneal Dialysis in Adults and Children [Internet]. Available from: https://renal.org/wp-content/uploads/2017/06/final-peritoneal-dialysis-guideline667ba231181561659443ff000014d4d8.pdf. [cited 2018 Apr 29].

[221] Rioux J-P, Marshall MR, Faratro R, Hakim R, Simmonds R, Chan CT (2015). Patient selection and training for home hemodialysis: Patient selection and training. Hemodial Int.

[222] Lockridge R, Cornelis T, Van Eps C (2015). Prescriptions for home hemodialysis: Prescriptions for home HD. Hemodial Int.

[223] Fotheringham J, Fogarty DG, El Nahas M, Campbell MJ, Farrington K (2015). The mortality and hospitalization rates associated with the long interdialytic gap in thrice-weekly hemodialysis patients. Kidney Int.

[224] Kraus MA, Kansal S, Copland M, Komenda P, Weinhandl ED, Bakris GL (2016). Intensive Hemodialysis and Potential Risks With Increasing Treatment. Am J Kidney Dis.

[225] Faratro R, Jeffries J, Nesrallah GE, MacRae JM (2015). The care and keeping of vascular access for home hemodialysis patients. Hemodial Int.

[226] Tong A, Palmer S, Manns B, Craig JC, Ruospo M, Gargano L (2013). The beliefs and expectations of patients and caregivers about home haemodialysis: an interview study. BMJ Open.

[227] Morfin JA, Fluck RJ, Weinhandl ED, Kansal S, McCullough PA, Komenda P (2016). Intensive Hemodialysis and Treatment Complications and Tolerability. Am J Kidney Dis.

[228] Achlarska M, Moon S (2017). Identifying and overcoming barriers to solo home haemodialysis. J Kidney Care.

[229] Liu N, Kim J, Jung Y, Arisy A, Nicdao MA, Mikaheal M, et al. Remote Monitoring Systems for Chronic Patients on Home Hemodialysis: Field Test of a Copresence-Enhanced Design. JMIR Hum Factors. 2017;4(3) Available from: https://www.ncbi.nlm.nih.gov/pmc/articles/PMC5596297/. [cited 2018 Apr 21]. 10.2196/humanfactors.7078 PMC5596297 28851680

[230] Elliott J, Rankin D, Jacques RM, Lawton J, Emery CJ, Campbell MJ (2015). A cluster randomized controlled non-inferiority trial of 5-day Dose Adjustment for Normal Eating (DAFNE) training delivered over 1 week versus 5-day DAFNE training delivered over 5 weeks: the DAFNE 5 x 1-day trial. Diabet Med.

[231] Cavanaugh KL, Wingard RL, Hakim RM, Eden S, Shintani A, Wallston KA (2010). Low health literacy associates with increased mortality in ESRD. J Am Soc Nephrol.

[232] Umeukeje EM, Merighi JR, Browne T, Victoroff JN, Umanath K, Lewis JB (2015). Self-Motivation Is Associated With Phosphorus Control in End-Stage Renal Disease. J Ren Nutr.

[233] Smith K, Coston M, Glock K, Elasy TA, Wallston KA, Ikizler TA (2010). Patient perspectives on fluid management in chronic hemodialysis. J Ren Nutr.

[234] Loud F, Gallagher H (2013). Kidney Health: Delivering Excellence. In: Group. TKHA, editor.

[235] World Health Organisation. Available from: http://www.who.int/gpsc/5may/en/. Accessed June 2018.

[236] Glidewell L, Boocock S, Pine K, Campbell R, Hackett J, Gill S (2013). Using behavioural theories to optimise shared haemodialysis care: a qualitative intervention development study of patient and professional experience. Implement Sci.

[237] Keith DS, Nichols GA, Gullion CM, Brown JB, Smith DH (2004). Longitudinal follow-up and outcomes among a population with chronic kidney disease in a large managed care organization. Arch Intern Med.

[238] Kato A, Odamaki M, Yamamoto T, Yonemura K, Maruyama Y, Kumagai H, Hishida A (2003). Influence of body composition on 5 year mortality in patients on regular haemodialysis. Nephrol Dial Transplant.

[239] Stack AG, Molony DA, Rives T, Tyson J, Murthy BV (2005). Association of physical activity with mortality in the US dialysis population. Am J Kidney Dis.

[240] Johansen KL, Chertow GM, Ng AV, Mulligan K, Carey S, Schoenfeld PY, Kent-Braun JA (2000). Physical activity levels in patients on hemodialysis and healthy sedentary controls. Kidney Int.

[241] Cheema BS, Singh MA (2005). Exercise training in patients receiving maintenance hemodialysis: a systematic review of clinical trials. Am J Nephrol.

[242] Cheema BS, Chan D, Fahey P, Atlantis E (2014). Effect of progressive resistance training on measures of skeletal muscle hypertrophy, muscular strength and health related quality of life in patients with chronic kidney disease: A systematic review and meta-analysis. Sports Med.

[243] Segura-Orti E (2010). Exercise in haemodyalisis patients: a literature systematic review. Nefrologia.

[244] Heiwe S, Jacobson SH. Exercise training for adults with chronic kidney disease. Cochrane Database Syst Rev. 2011;(10):CD003236. 10.1002/14651858.CD003236.pub2 PMC10183198 21975737

[245] Heiwe S, Jacobson SH (2014). Exercise training in adults with CKD: a systematic review and meta-analysis. Am J Kidney Dis.

[246] Smart N, Steele M (2011). Exercise training in haemodialysis patients: a systematic review and meta-analysis. Nephrology (Carlton).

[247] Sheng K, Zhang P, Chen L, Cheng J, Wu C, Chen J (2014). Intradialytic exercise in hemodialysis patients: a systematic review and meta-analysis. Am J Nephrol.

[248] Phan K, Jia F, Kamper SJ (2016). Effects of regular physical exercise training in adults with chronic kidney disease (PEDro synthesis). Br J Sports Med.

[249] Kirkman DL, Roberts LD, Kelm M, Wagner J, Jibani MM, Macdonald JH (2013). Interaction between intradialytic exercise and hemodialysis adequacy. Am J Nephrol.

[250] Bennett PN, Breugelmans L, Agius M, Simpson-Gore K (2007). Barnard B.A haemodialysis exercise programme using novel exercise equipment: a pilot study. J Ren Care.

[251] Musavian AS, Soleimani A, Masoudi Alavi N, Baseri A, Savari F (2015). Comparing the effects of active and passive intradialytic pedaling exercises on dialysis efficacy, electrolytes, hemoglobin, hematocrit, blood pressure and health-related quality of life. Nurs Midwifery Stud.

[252] Orcy R, Antunes MF, Schiller T, Seus T, Böhlke M (2014). Aerobic exercise increases phosphate removal during hemodialysis: a controlled trial. Hemodial Int.

[253] Pellizzaro CO, Thomé FS, Veronese FV (2013). Effect of peripheral and respiratory muscle training on the functional capacity of hemodialysis patients. Ren Fail.

[254] Lowrie EG, Braun Curtin R, Le Pain N (2003). Medical outcomes study Short Form-36: a consistent and powerful predictor of morbidity and mortality in dialysis patients. Am J Kidney Dis.

[255] Watson AR, Thurlby D, Schröder C, Fischbach M, Schaefer F, Edefonti A, Stefanidis CJ, Rönnholm K, Zurowska A (2000). Choice of end stage renal failure therapy in eight European centres. Pediatr Nephrol.

[256] Bunchman TE (1996). Pediatric hemodialysis: lessons from the past, ideas for the future. Kidney Int.

[257] Watson AR (1995). Strategies to support families of children with end stage renal failure. Pediatr Nephrol.

[258] Watson AR, Shooter M, Khanna R (1996). Transitioning adolescents from paediatric to adult dialysis units. Adv Perit Dial Publications 12.

[259] Daugirdas JT, Depner TA, Greene T (2010). Standard Kt/Vurea: a method of calculation that includes effects of fluid removal and residual kidney clearance. Kidney Int.

[260] Albalate M, Ruiz-Alvarez MJ, de Sequera P, Perez-Garcia R, Arribas P, Corchete E, Ruiz Caro C, Talaván Zanón T, Alcazar R, Ortega M, Puerta M (2017). Follow a recipe to prescribe phosphate during hemodialysis. Nefrologia.

